# Human frataxin, the Friedreich ataxia deficient protein, interacts with mitochondrial respiratory chain

**DOI:** 10.1038/s41419-023-06320-y

**Published:** 2023-12-08

**Authors:** Davide Doni, Federica Cavion, Marco Bortolus, Elisa Baschiera, Silvia Muccioli, Giulia Tombesi, Federica d’Ettorre, Daniele Ottaviani, Elena Marchesan, Luigi Leanza, Elisa Greggio, Elena Ziviani, Antonella Russo, Milena Bellin, Geppo Sartori, Donatella Carbonera, Leonardo Salviati, Paola Costantini

**Affiliations:** 1 Department of Biology, University of Padova, 35121 Padova, Italy; 2 Department of Chemical Sciences, University of Padova, 35131 Padova, Italy; 3 Clinical Genetics Unit, Department of Women’s and Children Health, University of Padova, 35128 Padova, Italy; 4 Istituto di Ricerca Pediatrica (IRP) Città della Speranza, 35127 Padova, Italy; 5 Centro Studi per la Neurodegenerazione (CESNE), University of Padova, Padova, Italy; 6 Department of Molecular Medicine, University of Padova, 35121 Padova, Italy; 7 Veneto Institute of Molecular Medicine, 35129 Padova, Italy; 8 Department of Anatomy and Embryology, Leiden University Medical Center, 2333 ZA Leiden, The Netherlands; 9 Department of Biomedical Sciences, University of Padova, 35121 Padova, Italy

**Keywords:** Mitochondria, Mechanisms of disease

## Abstract

Friedreich ataxia (FRDA) is a rare, inherited neurodegenerative disease caused by an expanded GAA repeat in the first intron of the *FXN* gene, leading to transcriptional silencing and reduced expression of frataxin. Frataxin participates in the mitochondrial assembly of FeS clusters, redox cofactors of the respiratory complexes I, II and III. To date it is still unclear how frataxin deficiency culminates in the decrease of bioenergetics efficiency in FRDA patients’ cells. We previously demonstrated that in healthy cells frataxin is closely attached to the mitochondrial cristae, which contain both the FeS cluster assembly machinery and the respiratory chain complexes, whereas in FRDA patients’ cells with impaired respiration the residual frataxin is largely displaced in the matrix. To gain novel insights into the function of frataxin in the mitochondrial pathophysiology, and in the upstream metabolic defects leading to FRDA disease onset and progression, here we explored the potential interaction of frataxin with the FeS cluster-containing respiratory complexes I, II and III. Using healthy cells and different FRDA cellular models we found that frataxin interacts with these three respiratory complexes. Furthermore, by EPR spectroscopy, we observed that in mitochondria from FRDA patients’ cells the decreased level of frataxin specifically affects the FeS cluster content of complex I. Remarkably, we also found that the frataxin-like protein Nqo15 from *T. thermophilus* complex I ameliorates the mitochondrial respiratory phenotype when expressed in FRDA patient’s cells. Our data point to a structural and functional interaction of frataxin with complex I and open a perspective to explore therapeutic rationales for FRDA targeted to this respiratory complex.

## Introduction

Friedreich ataxia (FRDA; OMIM 229300) is an autosomal recessive neurodegenerative disorder clinically characterized by slowly progressive gait and limb ataxia, areflexia, dysarthria and loss of proprioceptive sensation [1–3]. Symptoms commonly appear during childhood or adolescence, with loss of coordination, muscle weakness and fatigue, and gradually lead patients to motor incapacitation and wheelchair reliance 15–20 years after the onset of the disease. Although FRDA typically afflicts the nervous system, nearly two thirds of all patients present cardiac impairment or myocardium thickening [4] and compensatory hypertrophic cardiomyopathy is the primary cause of death, in the 3rd to 5th decade of life [3–5]. Ninety-five percent of FRDA patients are homozygous for a GAA triplet expansion in the first intron of the *FXN* gene; this leads to transcriptional silencing of *FXN* through heterochromatinization of the expanded region and to reduced expression of frataxin, a ubiquitous and highly conserved mitochondrial protein [1, 6, 7]. The severity of the disease increases with the length of the expansion, which ranges from 6–36 triplets in healthy individuals to 70–1700 triplets in FRDA patients [1]. Approximately 5% of the patients are compound heterozygous for the GAA expansion and a different mutation resulting in the loss of stability and/or function of the protein [8, 9]. All patients have about 5–30% estimated residual frataxin levels, depending on the extent of the silencing [6, 10]. Complete loss of frataxin is not compatible with life, as indicated by the embryonic lethality upon inactivation of *FXN* in mice [11].

To date, the medical care of FRDA patients is essentially aimed at alleviating symptoms, but the research in the field has advanced considerably in recent years and several potential drugs are currently in various stages of development [12]. Current therapeutic strategies aim to increase frataxin levels or mitigate the late consequences of its deficiency (www.curefa.org/research/research-pipeline). In this respect, it is worth noting that the U.S. Food and Drug Administration has recently approved omaveloxolone, a molecule targeting the Nrf2-antioxidant pathway, as a drug to mitigate the oxidative damages occurring as a secondary effect of mitochondrial dysfunctions in FRDA patients [13]. The mechanisms underlying the disease are not fully understood and in the search for further effective drugs it is essential to understand how frataxin deficiency precipitates the patient’s cellular phenotype, which is still unclear. Frataxin plays a role in the mitochondrial biogenesis of FeS clusters [14–17], small metallocofactors which contribute to several cell functions, including redox catalysis, β-oxidation of lipids, gene expression regulation, and DNA repair/replication [18]. They are crucial in the mitochondrial oxidative phosphorylation, since they move electrons through respiratory complexes I, II and III. Consistently, several human diseases are caused by a defective FeS clusters biogenesis [19, 20]. Frataxin was shown to interact in vitro with the mitochondrial FeS cluster assembly machinery [21–24]. The contribution of frataxin to the synthesis of the FeS clusters is still under debate, however a consensus exists pointing to a function as sulfide production stimulator or accelerator of sulfur transfer from cysteine [25–30]. It is worth noting that FRDA models and patients’ cells show impaired cellular respiration and reduced ATP production [31–39], which is particularly relevant in energy-intensive cells, as neurons and cardiomyocytes.

We have previously found that in healthy cells frataxin, despite being a soluble protein lacking transmembrane domains [40, 41], is closely attached to the mitochondrial inner membrane and enriched in the cristae [42], which contain both the FeS cluster assembly machinery [42, 43] and the respiratory chain complexes. On the contrary, residual frataxin is largely displaced in the matrix in cells from FRDA patients with impaired respiration [42]. This indicates that whatever the specific physiological role of frataxin in the mitochondrial phenotype, it relies on its enrichment in the cristae, where the interactions with potential partners should be facilitated.

It was shown that the *Saccharomyces cerevisiae* frataxin orthologue Yfh1p interacts with the respiratory complex II subunits Sdh1p and Sdh2p, which bind the FAD cofactor and three FeS clusters, respectively [44]. In the same work, an interaction of human frataxin with complex II was found by co-immunoprecipitation experiments using the recombinant proteins expressed in yeast. Interestingly, it was also shown that the deletion of the frataxin homologue in *Escherichia coli* led to a decrease of the complex I and complex II content by approximately one third and one quarter, respectively [45]. Intriguingly, the crystal structure of the peripheral arm of the complex I from *Thermus thermophilus* revealed the presence of a subunit (i.e., Nqo15) sharing a highly similar three-dimensional fold with both prokaryotic and eukaryotic frataxin proteins, despite the low sequence similarity [46, 47]. Taken together, these data suggest that human frataxin could interact with the mitochondrial respiratory chain as well, and that its decrease in FRDA cells could have an impact at this level.

To gain novel insights into the function of frataxin and in the events leading to the bioenergetics defects afflicting FRDA patients, in this work we explored the close proximity of frataxin with the respiratory chain in healthy cells and in different cellular models of the disease.

## Results

### Human frataxin interacts with the mitochondrial respiratory chain

We first addressed the issue of whether human frataxin can interact with the respiratory chain. Three of the four respiratory complexes (i.e., CI, CII and CIII) heavily rely on FeS clusters to move electrons, and their redox activity turned out to be affected in several FRDA cellular models [31, 42, 48].

**Fig. 1:**
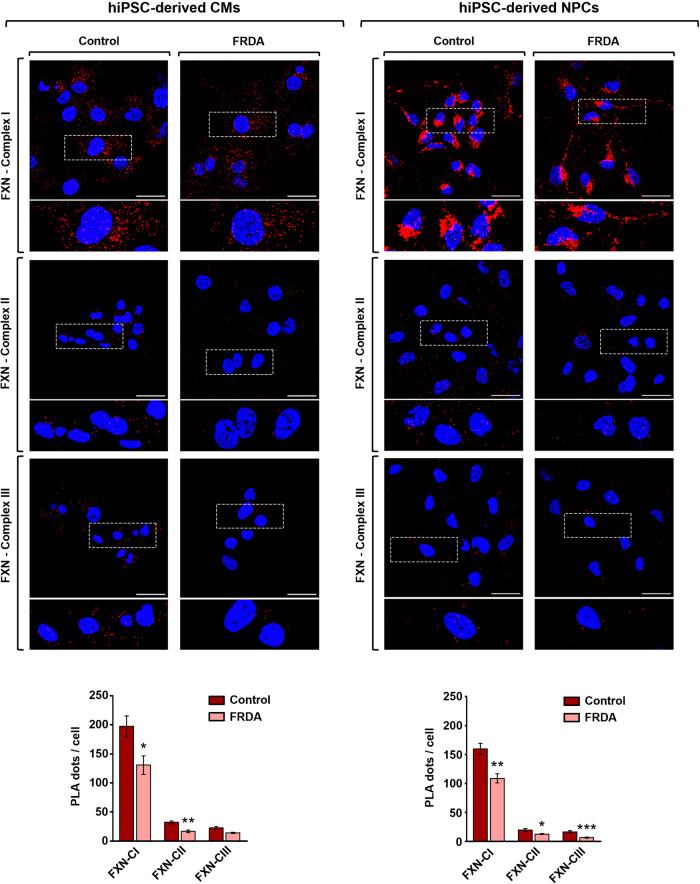
Frataxin is in close proximity to mitochondrial respiratory chain complexes I, II and III in healthy hiPSC-derived CMs and hiPSC-derived NPCs. On the top of both panels, representative confocal images of in situ proximity ligation assay (PLA) performed in hiPSC-CMs (left panel) and hiPSc-NPCs (right panel) using an anti-frataxin antibody in combination with antibodies against the respiratory chain complexes I, II or III. Scale bar: 30 μm. Nuclei were counterstained with DAPI (blue). Red fluorescent dots were quantified and reported as PLA dots/cell (on the bottom). Analyzed data, reported as the means ± SEM, derived from three independent experiments in which at least five different fields were acquired. Statistical significance was determined using unpaired *t*-test (*p ≤ 0.05, **p ≤ 0.01, ***p ≤ 0.001 compared to healthy control cell lines).

**Fig. 2:**
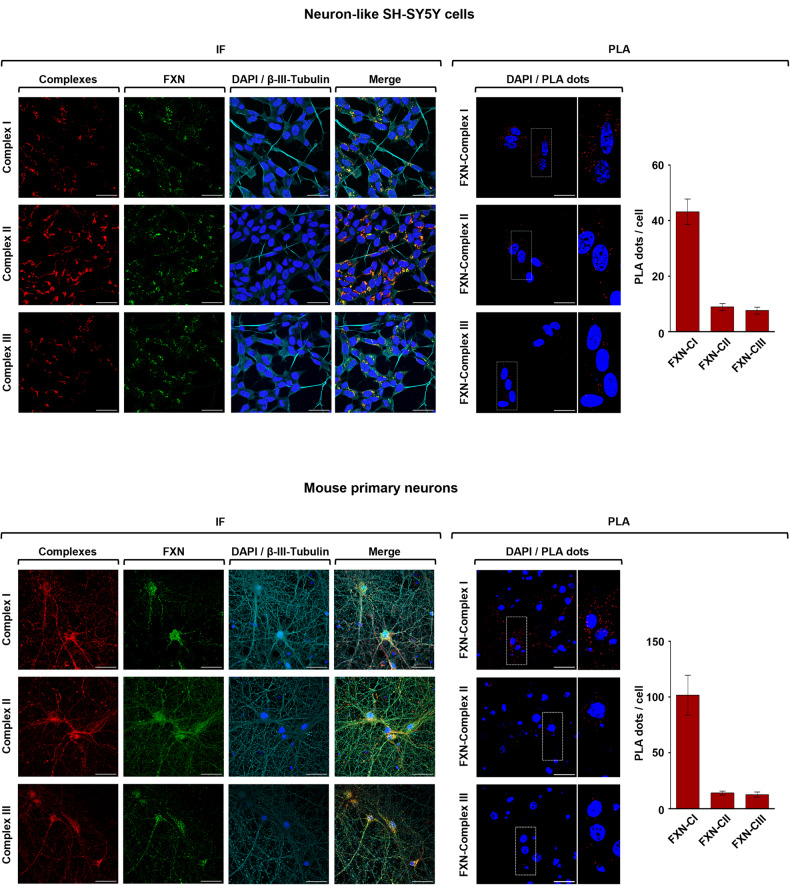
Frataxin proximity to mitochondrial respiratory chain is confirmed in neuron-like SH-SY5Y cells and in mouse primary neurons. Representative PLA confocal images confirming the co-localization and the physical proximity of FXN with respiratory complexes I, II, or III in healthy retinoic acid-differentiated neuroblastoma SH-SY5Y cells (upper panels) and mouse primary neurons (lower panels). On the left of each panel, double immunofluorescence staining shows in green FXN, in red respiratory complexes I, II, or III and in yellow the merge of fluorescent signals. β-III-Tubulin was chosen as neural marker (cyan). On the right, red fluorescent dots indicate the closeness of FXN to respiratory complexes I, II and III as assessed by in situ PLA. In both IF and PLA images, nuclei were counterstained with DAPI (blue). Scale bars: 30 μm. Red fluorescent dots were quantified and reported as PLA dots/cell. Analyzed data, reported as the means ± SEM, derived from three independent experiments in which at least five different fields were acquired.

The closeness of frataxin to respiratory complexes I, II and III was analyzed by the Proximity Ligation Assay (PLA), that enables the detection of proximity between couples of proteins in situ (at distances <40 nm), visualized as discrete fluorescent dots by confocal microscopy. Since neurological and cardiac involvement dominate the clinical picture of FRDA, we performed PLA in cardiomyocytes (CMs) and neural progenitor cells (NPCs) derived from human induced pluripotent stems cells (hiPSCs) (which have been shown to faithfully recapitulate some of the structural and functional defects associated with FRDA [49–56]) generated from a healthy control and from a FRDA patient clinically affected at sampling, with spinal-cerebral degeneration and cardiomyopathy (Supplementary Fig. 1). At first, by immunofluorescence analysis we confirmed the mitochondrial co-localization of frataxin and the respiratory complexes I, II and III (Supplementary Fig. 2). As expected, the fluorescence signal associated to frataxin was decreased in FRDA CMs and NPCs, in which the expression of the protein is reduced (Supplementary Fig. 3). The same combination of antibodies was used for the PLA experiments. As shown in Fig. 1, fluorescent dots indicate the proximity of frataxin to respiratory complexes I, II and III in healthy CMs and NPCs (left columns in each panel). Negative control experiments, carried out by omitting either the primary or the secondary antibodies, resulted in the complete absence of PLA dots (Supplementary Fig. 4). The specificity of the approach was further confirmed by the reduction of PLA signals in FRDA CMs and NPCs (Fig. 1, right columns in each panel), in accordance with the frataxin decrease. The quantitative analysis of fluorescent dots (i.e., number of dots/cell) resulted in a higher number with the couple of antibodies anti-frataxin/anti-complex I, both in CMs and NPCs. The same trend was found in SH-SY5Y human neuroblastoma-derived neuron-like cells and mouse primary neurons, two cellular models widely used in experimental neurological studies (Fig. 2).

**Fig. 3:**
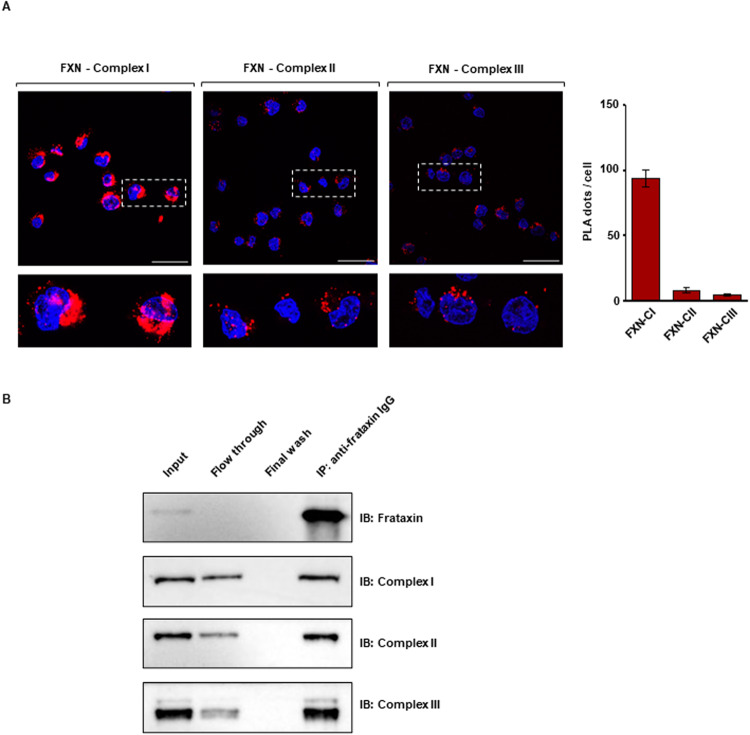
Human frataxin interacts with respiratory chain complexes I, II and III in healthy LCLs. **A** Representative confocal images of in situ PLA experiments performed as described in the legend of Fig. 1. **B** Western blotting analysis of co-immunoprecipitation (co-IP) experiments (representative image of one of three independent experiments). Input corresponds to the total cell lysate used for co-IP assay; flow through and final wash represent the fractions eluted from the incubation of cell lysate with the resin and collected after the third wash, respectively. The supernatant containing the IP sample after denaturation of the resin was loaded in the fourth lane. Equal volumes (i.e., 35 μL) of samples were loaded in each lane. Blots were immunodecorated with antibodies against FXN, NDUFS1 (complex I), SDHB (complex II) and UQCRFS1 (complex III).

Taken together, these data indicate that frataxin and the respiratory complexes are close enough to allow protein-protein interactions, that we next investigated by co-immunoprecipitation experiments. We switched to lymphoblastoid cells (LCLs), which have higher growth rates compared to hiPSCs-CMs and hiPSCs-NPCs and allowed to obtain the required yield in terms of endogenous protein concentration. First, we confirmed by PLA analysis the proximity of frataxin to the complexes I, II and III, with a prevalence of fluorescent dots around complex I (Fig. 3A). Immunoprecipitation of a whole cell extract using an anti-frataxin antibody indicated that frataxin interacts with complexes I, II and III (Fig. 3B).

### FeS clusters content of respiratory complex I is decreased in FRDA patients’ cells

To fulfill their function in electron channeling through the respiratory chain, complexes I, II and III are equipped with several FeS clusters in their core subunits (eight, three and one, respectively) [57]. To gain insights into the consequence of frataxin decrease at the level of the FeS clusters in FRDA patients’ cells, we used Electron Paramagnetic Resonance (EPR) spectroscopy to evaluate the content of these cofactors in intact mitochondria from healthy control (same line used for the co-immunoprecipitation experiments) and two FRDA LCLs homozygous for the GAA triplet expansion (that we named FRDA1 and FRDA2). The alteration of the mitochondrial respiratory phenotype of these cells has been thoroughly characterized in our laboratory, showing a decrease of the oxygen consumption rate [42], which makes them a good model to interpret the results of EPR analyses in the context of bioenergetics defects in FRDA.

**Fig. 4:**
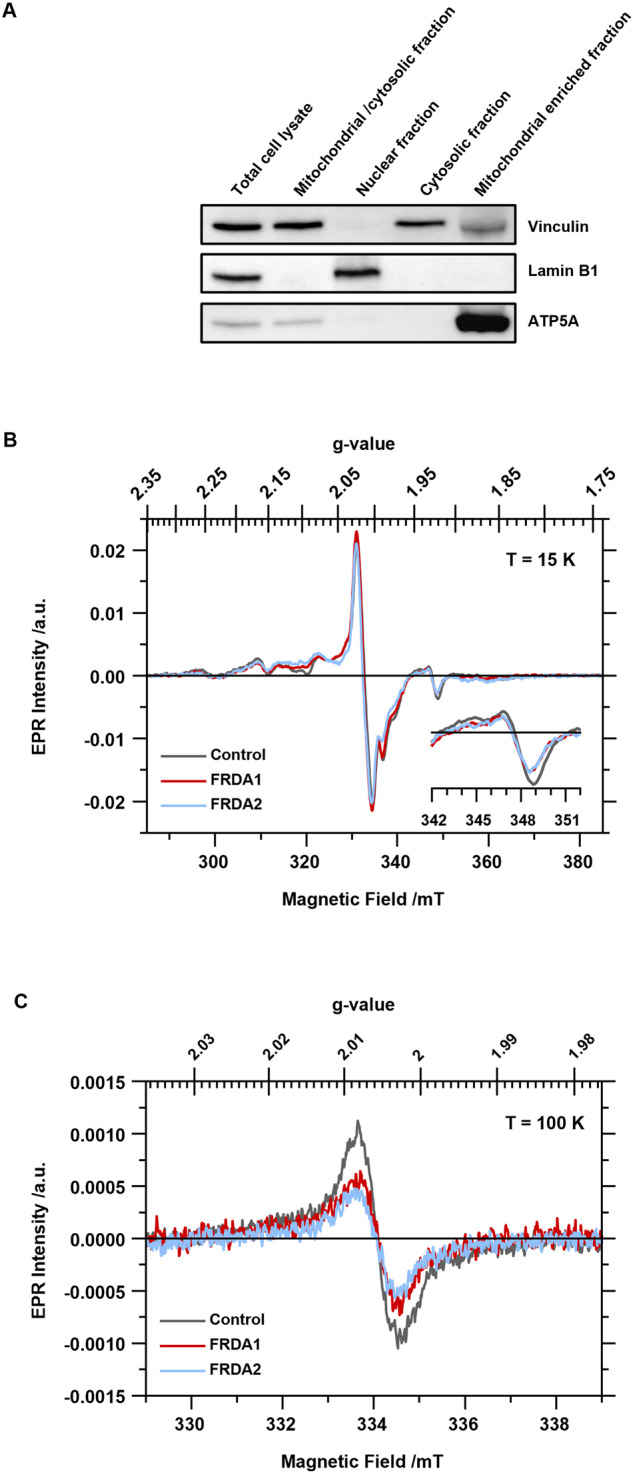
Evaluation of paramagnetic cofactors from healthy and FRDA LCLs’ mitochondria by EPR spectroscopy. **A** Western blotting analysis for the subcellular fractionation of LCLs. Cells were fractionated to nuclear, cytosolic and mitochondrial fractions by sequential centrifugation. Equal volumes of the samples (i.e., 5 μL) were loaded in each lane. Vinculin, lamin B1 and ATP5A have been chosen as cytosolic, nuclear and mitochondrial markers, respectively. The reported western blot refers to the subcellular fractionation of the healthy LCLs used in this work. **B** EPR spectra acquired at 15 K of the mitochondrial enriched fractions from LCLs. The superimposed spectra are reported with a different color for each cell line: dark gray, control; red, FRDA1; light blue, FRDA2. The inset on the spectra at 15 K refers to the signal of N2 cluster from complex I magnified by factor of 8. **C** EPR spectra acquired at 100 K, colors as in **B**.

The EPR spectra of the mitochondria, as purified from the three cell lines by subcellular fractionation (Fig. 4A), without further chemical treatment, were recorded at 15 and 100 K (Fig. 4). The spectra at 15 K were typical for whole mammalian mitochondria quickly frozen after isolation [58]. Several signals from the paramagnetic cofactors of the respiratory chain complexes were present (reference [59] reports a comprehensive overview of the different mitochondrial EPR signals). Most interesting for our investigation is that the EPR signal from complex I (peak-to-peak intensity of the isolated peak of the N2 cluster at g = 1.92, inset in Fig. 4B) decreased in patients’ mitochondria relative to the control (FRDA1 −25 ± 5%; FRDA2 −29 ± 5%), while the overall intensity of the complex II + complex IV signal (peak-to-peak intensity of the central feature, i.e*.* the sum of S3 [3Fe-4S] cluster and the SQ-SQ signal from the semiquinone pair in complex II and the perpendicular axial component of CuA) was unchanged in patients’ mitochondria relative to the control (FRDA1 + 5 ± 5%; FRDA2 −3 ± 5%). At 100 K, the EPR spectra quantify the overall signal of radical cofactors, semiquinones and semiflavines (i.e. coenzyme Q10 and FMN from complex I, FAD from complex II other quinones from complex III). The radical species decrease in patients’ mitochondria relative to the control (Fig. 4C - FRDA1 −37 ± 10%; FRDA2 −52 ± 10%). The radical signals have long been attributed mainly to complex I, therefore we could link the decrease directly to a loss of complex I; however, recently it has been suggested that in isolated mitochondria the majority of the radical signal arises from semiquinones located in complex III [60], and thus caution should be exercised.

**Fig. 5:**
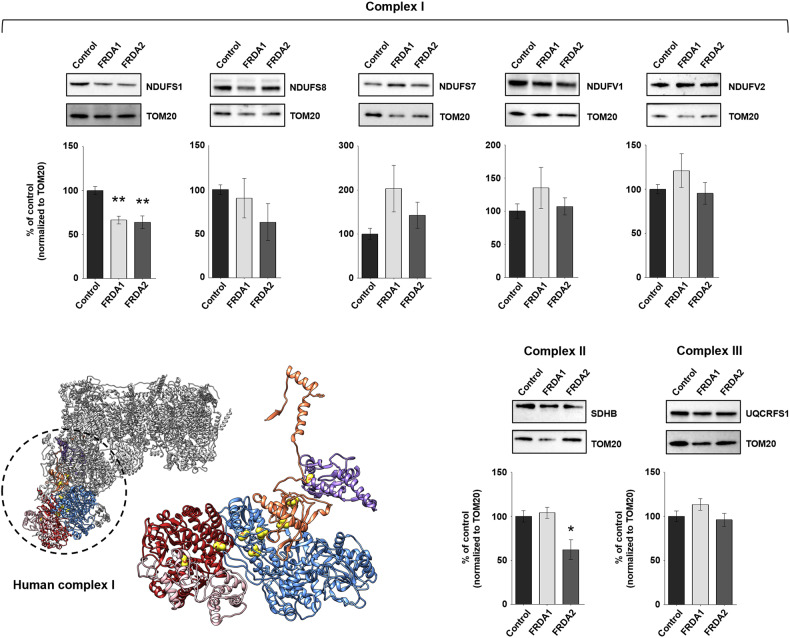
Comparative analysis of respiratory complexes I, II and III subunits containing FeS clusters in healthy and FRDA LCLs. Western blotting analysis of respiratory complexes subunits containing FeS clusters, i.e., NDUFS1, NDUFS8, NDUFS7, NDUFV1, NDUFV2 (complex I), SDHB (complex II) and UQCRFS1 (complex III), in whole cells extracts from FRDA (1 and 2) and healthy LCLs. Equal amounts of protein (i.e., 25 µg) were loaded in each lane. TOM20 was used as mitochondrial loading control. Protein levels were quantified after normalization with TOM20 and expressed as a percentage of control level (healthy LCLs). Reported data result from the mean of at least six independent experiments ± SEM. Statistical significance was determined using One-way ANOVA with Dunnett’s post-hoc test (*p ≤ 0.05, **p ≤ 0.01, compared to control). Figure reports the cartoon representation of human respiratory complex I structure (PDB ID: 5XTD) in which the subunits containing FeS clusters are highlighted in different colors (NDUFS1: light blue; NDUFS8: orange; NDUFS7: purple; NDUFV1: red; NDUFV2: pink). Iron and sulfur atoms belonging to FeS clusters are shown as brown and yellow spheres, respectively. To better visualize the number and position of FeS clusters, the subunits containing these cofactors were zoomed and reoriented on the right of the structural representation.

Since the loss of EPR intensity can be attributed either to a loss of protein, or to a lower degree of reduction of the cofactors, we next examined by western blotting the relative abundance of respiratory complexes I, II and III core subunits containing FeS clusters, in the control and in the two FRDA LCLs: for complex I, NDUFV1, NDUFV2, NDUFS1, NDUFS7 and NDUFS8 (which collectively contain eight FeS clusters); for complex II, SDHB (which contains three FeS clusters); for complex III, UQCRFS1 (which contains a single FeS cluster). For the sake of clarity, the position of the subunits selected for complex I as well as their FeS clusters are shown in Fig. 5. The comparative analysis of the levels of these subunits in control and FRDA LCLs, normalized to TOM20 as mitochondrial protein loading control, produced variable results, depending on the cell line (Fig. 5). We found that the expression levels of some subunits of complex I, i.e*.*, NDUFS1 and NDUFS8, decreases in both FRDA LCLs, while the others are unchanged or slightly increased. Complex II SDHB protein level is unaltered in FRDA1 and reduced in FRDA2 LCLs. Complex III UQCRFS1 levels are unaffected in both FRDA LCLs.

### Expression of the frataxin-like Nqo15 subunit of complex I from *T. thermophilus* increases mitochondrial respiration in FRDA patient’s cells

**Fig. 6:**
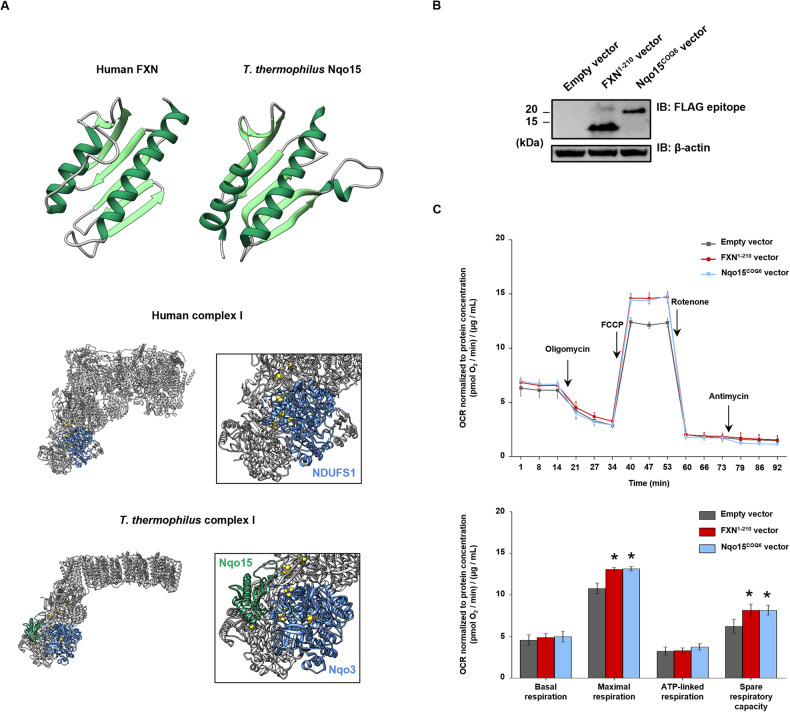
Cellular respiration of FRDA fibroblasts expressing the frataxin-like protein Nqo15 of *T. thermophilus* complex I. **A** On the top, cartoon representation of human mature frataxin (PDB ID: 1EKG) and the subunit Nqo15 from *T. thermophilus* respiratory complex I (PDB ID: 6Y11). In both structures, α helices and β strands are highlighted in dark and light green, respectively. On the bottom, structural representation of respiratory complex I from human mitochondria (PDB ID: 5XTD) and *T. thermophilus* (PDB ID: 6Y11). Iron and sulfur atoms of FeS clusters are shown as brown and yellow spheres, respectively. For sake of clarity, the domain of *T. thermophilus* complex I containing Nqo15 (in green) and the corresponding region in the human structure have been zoomed. In the bacterial complex, Nqo15 faces the subunit Nqo3 which corresponds to the homologue subunit NDUFS1 in human complex I (both subunits are shown in light blue). **B** Western blotting analysis of the whole protein extract obtained 72 h after lentiviral transduction of FRDA fibroblast with Nqo15^COQ6^ vector or FXN^1-210^ vector. As negative control, cells were infected with the empty vector. The expression of the exogenous proteins was detected immunodecorating the blot with an anti-FLAG primary antibody. Equal volumes of sample in each lane (i.e., 20 µL) were loaded and β-actin was used as loading control. **C** Oxygen consumption rates (OCR) of cells were measured in real time under basal conditions and after injection of oligomycin (1 µM), FCCP (0.75 µM), rotenone (1 µM) and antimycin (1 µM) as indicated in the figure. Values were normalized to the total protein concentration (µg/mL) and the respiration profiles of the transduced fibroblasts are represented in dark gray for negative control, in red for FXN^1-210^ vector and in light blue for Nqo15^COQ6^ vector. Bioenergetic parameters were calculated as described in Materials and Methods. Reported data result from the mean of three independent experiments ± SEM. Statistical significance was determined using Two-way ANOVA with Bonferroni’s post-hoc test (*p ≤ 0.05, compared to empty vector transduction).

In addition to the 14 core subunits conserved from bacteria to mammals [57], the hydrophilic domain of complex I from *T. thermophilus* contains a further subunit, called Nqo15 [46, 47]. Interestingly, despite the low sequence similarity (i.e., 11% of identity), the three-dimensional fold of Nqo15 superimposes very well with that of human frataxin, which consists of a twisted, six-stranded antiparallel β sheet, flanked on one side by two α helices (Fig. 6A). This prompted us to express this frataxin-like protein in FRDA patient’s cells and to evaluate the potential effect on mitochondrial respiration. To this end, we generated two lentiviral vectors containing *i)* the Nqo15 sequence fused at the 5’-terminus to the mitochondrial targeting signal of human COQ6, which has been previously used in our laboratory to address recombinant proteins into mitochondria, and *ii)* human frataxin sequence (FXN^1-210^), as a positive control in functional experiments. A FLAG epitope was also added at the 3’ of each construct to allow western blotting analyses. Lentiviral particles carrying the sequences of interest were obtained as described in Materials and Methods and used to individually transduce fibroblasts from a FRDA patient. As a negative control, cells were also infected with a viral empty vector under the same experimental conditions. By immunoblotting with an anti-FLAG antibody we verified that transduced cells express the recombinant proteins (Fig. 6B). In these cells, oxygen consumption rate (OCR) was measured by means of a Seahorse flux analyzer. Both maximal respiration and spare respiratory capacity increased in FRDA cells transduced with FXN, as expected, and, to same extent, in those transduced with Nqo15, compared to the cells transduced with the empty vector (Fig. 6C). Overall, these data indicate that the respiration of FRDA cells is improved upon expression of the frataxin-like protein Nqo15 from *T. thermophilus* complex I.

## Discussion

Despite several years of intense research, which led to important breakthroughs in exploring frataxin function, its specific role in cell physiology is still elusive. In FRDA patients, due to the deficit in frataxin, dysfunctional mitochondria cannot provide the required energy to high-demanding cells; the tissues mostly affected by frataxin decrease are indeed the dorsal root ganglia, spinal cord, cerebellar dentate nuclei, skeletal muscle, pancreas, and heart [61]. The involvement of frataxin in the early steps of the mitochondrial biogenesis of FeS clusters is widely accepted [21–24]. In this respect, mitochondria must be considered as double-sided: they are the major source of FeS clusters, and they heavily rely on these cofactors to fulfill their function in ATP production. Because of this sort of loop, it is difficult to clarify with certainty the primary defects leading to the mitochondrial bioenergetic failure of frataxin deficient cells, and this makes it challenging to identify potential therapies or specific drug targets for FRDA.

**Fig. 7:**
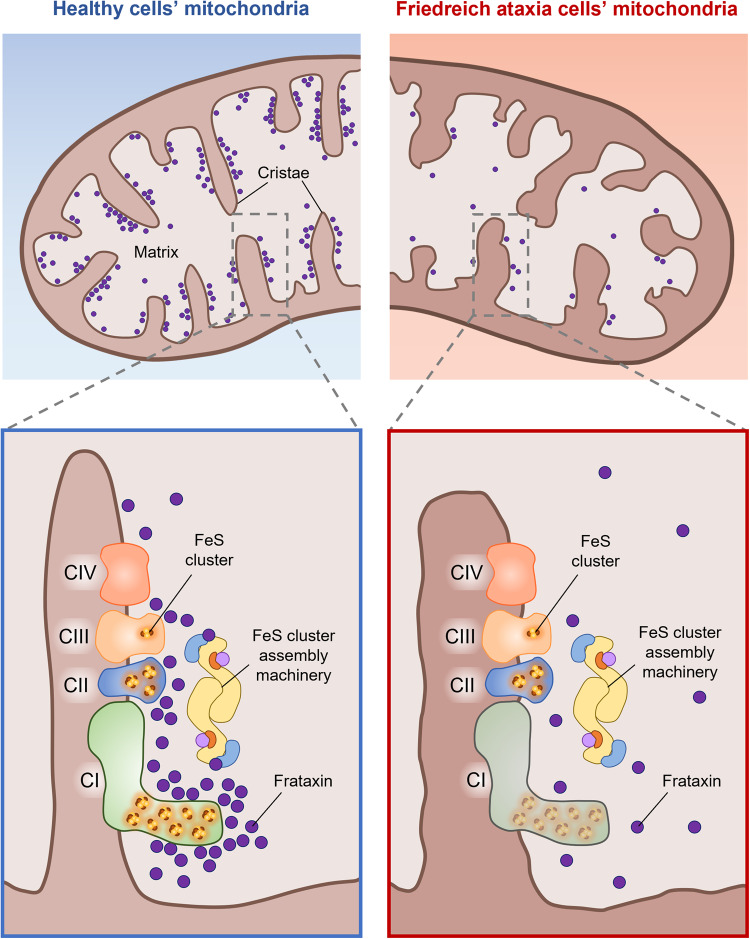
Graphic representation of the hypothesis explored in this work: a proof-of-concept that the loss of interaction between frataxin and mitochondrial respiratory complex I could be critical for the upstream bioenergetics’ defects in cells of patients with Friedreich ataxia. In healthy cells frataxin is closely attached to the mitochondrial cristae, which contain both the FeS cluster assembly machinery and the respiratory chain complexes; in FRDA cells with bioenergetics defects, perturbation of mitochondrial ultrastructure causes the mislocalization of the residual frataxin from the cristae membrane to the matrix. The enrichment of frataxin in the cristae in healthy cells makes possible its functional interaction with complex I (red box), which is lost in FRDA patients’ cells, resulting in the impairment of complex I (blue box).

We have previously shown that in healthy cells frataxin is enriched in the mitochondrial cristae [42], and it is therefore in proximity to the respiratory chain, which contains twelve FeS clusters distributed among complexes I, II and III. We also found that in FRDA cells with defects in mitochondrial ultrastructure and respiratory function, residual frataxin is widely spread in the matrix [42]. This prompted us to explore the hypothesis that frataxin interacts with the respiratory complexes containing FeS clusters, and that its enrichment in the cristae, essential to drive functional interactions with the partners, could be lost in FRDA cells with damaged mitochondria (as depicted in Fig. 7). In this work, by coupling co-immunoprecipitation and PLA experiments, we showed that human frataxin interacts with respiratory complexes I, II and III (Figs. 1–3). The PLA approach is highly specific because the fluorescent signals are only produced if the two proteins of interest are closer than 40 nm. Moreover, the number of dots of proximity/cell can be objectively quantified: the lower the distance between the two proteins, the higher the probability to obtain fluorescent dots, the higher the number of dots. A close inspection of the PLA images showed that the dots obtained with the couple of antibodies anti-frataxin/anti-complex I appear more clustered and are greater in number compared to those obtained with the anti-complex II or anti-complex III antibodies, in all cell lines used in this work. Frataxin is a soluble protein that is expected to diffuse freely between the hydrophilic portions of respiratory chain complexes: the results of PLA analysis indicate that the probability that frataxin is close enough to the respiratory complexes to allow a physical interaction is statistically higher with complex I. If this were the case, the major effect of its decrease on respiratory complexes, if any, would be expected for this complex.

The EPR spectroscopy of mitochondria purified from healthy and FRDA patients’ cells, provided interesting results. To the best of our knowledge this is the first attempt to detect the variations in FeS and other paramagnetic cofactors directly in the mitochondria of FRDA cells, in which we found that the EPR signals of cofactors belonging to complex I are significantly reduced relative to the control (Fig. 4): the signal from cluster N2 decreases, as the sum of all radical signals does, most of which have been traditionally attributed to complex I. In contrast, the overall variation of the cofactors from the other respiratory complexes is not significant, pointing to a specific susceptibility of complex I to frataxin decrease.

Complex I is the main entry site for electrons into the respiratory chain, and its largest component; it consists of 14 core subunits, which harbor all its redox centers [62–65], and 31 supernumerary subunits, without a direct catalytic function and involved in the assembly and stability of the complex [66]. All core subunits are evolutionarily conserved from bacteria to the mitochondria of higher eukaryotes [56, 67, 68]. Interestingly, complex I from *T. thermophilus* possess an additional core subunit, Nqo15, with a three-dimensional fold superimposing to the unique fold of frataxins [46, 47] (Fig. 6A). Nqo15 is bound to the side of the complex, where it interacts with the subunits Nqo3, Nqo2, Nqo1, Nqo9, and Nqo4, all containing FeS clusters except for Nqo4 which is at the interface of the FeS cluster binding site of Nqo6 [46, 69]. The role of Nqo15 in *T. thermophilus* complex I has not yet been clarified, however some hypothesis have been advanced [46]. First, in the absence of Nqo15 the structure of complex I would be rather narrow and likely unstable, hence one putative function could be the stabilization of the complex. Moreover, by interacting through its β-sheet with the side of complex I, Nqo15 allows the formation of a hydrophilic channel leading from the solvent to the region accommodating the FeS clusters N1a and N3 [46]. This channel is lined with six exposed histidine residues (four of them provided by Nqo15), that could form a possible binding surface for iron, which is also present in frataxins [24, 70–72]. Therefore, another possible role of Nqo15 could be the storage of iron for the assembly/regeneration of FeS clusters of complex I.

A relevant finding described herein is that the expression of a recombinant Nqo15 in FRDA patient’s cells led to an increase of the oxygen consumption rate (Fig. 6B, C), that, based on the structural similarity between frataxin and Nqo15, could indirectly suggest a functional interaction of human frataxin with the mitochondrial respiratory complex I. Of note, we found a significant decrease of the NDUFS1 subunit, the homologue of *T. thermophilus* Nqo3 which faces Nqo15 (Fig. 6A), in all FRDA patients’ cells used in this work (Fig. 5 and Supplementary Fig. 5). Therefore, as previously hypothesized for Nqo15, frataxin could stabilize complex I through a physical interaction with the side region of its peripheral arm, which accommodates the subunits containing all the FeS clusters wiring the electron flow and is particularly prone to intrinsic damage [73]. From a functional perspective, a free and rapid access to frataxin would be pivotal for the assembly and/or regeneration of complex I FeS clusters. In human cells the near totality of complex I appears associated with supercomplexes, quaternary structures which increase the structural stability of respiratory complexes and the overall mitochondrial efficiency in ATP production [74, 75]. The formation of supercomplexes starts only when complex I is fully assembled [76], and therefore any structural damage of complex I is expected to have an impact on this process. Interestingly, we had previously shown that the relative abundance of complex I-containing supercomplexes is decreased in FRDA patients’ cells compared to healthy cells [42]. This further supports the present results, which point to a structural and functional impairment of complex I in cells with reduced levels of frataxin.

Any insight into the physiological function of frataxin would be helpful to clarify the primary cause-effect relationship between frataxin decrease and bioenergetics failure and to move toward development/identification of drugs that specifically target the impaired mitochondrial functions of FRDA patients’ cells. Our findings shed new light on the role of frataxin in the metabolic dysfunctions leading to FRDA and could pave the way to future therapeutic strategies in the disease treatment focused on respiratory complex I. Experiments are currently underway in our laboratory to obtain molecular details of the interaction between frataxin and complex I that could drive the design of peptidomimetics able to stabilize this complex in FRDA patients’ cells.

## Materials and methods

### Cell lines and culture conditions

The following cell lines were obtained from the Human Genetic Cell Repository at the Coriell Institute for Medical Research (NJ, USA): lymphoblastoid cells (LCLs) GM04079 (*FXN* alleles of 541 and 420 repeats at sampling), GM16227 (*FXN* alleles of 630 and 830 repeats at sampling), GM07533 (both *FXN* alleles in the normal range of GAA repeats); fibroblasts GM04078 (*FXN* alleles of 541 and 420 repeats at sampling); human induced Pluripotent Stem Cells GM23404*B, reprogrammed from fibroblasts with *FXN* alleles of 338 and 380 repeats at sampling. Lymphoblastoid cell lines GM04079 and GM16227 are indicated throughout the text as FRDA 1 and FRDA 2, respectively. Control fibroblasts from a healthy subject were available in our laboratory. Control hiPSC line was from a healthy subject: LUMC0020iCTRL-06 (https://hpscreg.eu/cell-line/LUMCi028-A) [77]. The SH-SY5Y neuroblastoma cell line was purchased form the Cell Factory-IST Genova. All cell lines used in this work were routinely tested for mycoplasma and resulted negative.

LCLs were cultured in RPMI1640 (GIBCO Life Technologies) with the addition of 2 mM L-glutamine and supplemented with 15% (v/v) FBS (GIBCO Life Technologies), 100 U/mL penicillin and 100 μg/mL streptomycin (GIBCO Life Technologies), under 5% CO_2_ at 37 °C. Fibroblasts were cultured in DMEM high-glucose (GIBCO Life Technologies) supplemented with 2 mM L-glutamine, 10 μM uridine, 100 mM sodium pyruvate (100X solution, GIBCO Life Technologies), 20% (v/v) FBS (GIBCO Life Technologies), 100 U/mL penicillin, 100 µg/mL streptomycin, under 5% CO_2_ at 37 °C. For cell passaging, fibroblasts were washed with Phosphate Buffered Saline (PBS), detached with 0.25% trypsin/EDTA (GIBCO Life Technologies) and seeded with fresh growth medium. SH-SY5Y neuroblastoma cells were cultured for 7 days under 5% CO_2_ at 37 °C in differentiation medium composed of a mixture (1:1) of DMEM (Biowest) and Ham’s F-12 (Biowest) and supplemented with 1% (v/v) FBS (ThermoFisher Scientific), 100 U/mL penicillin, 100 μg/mL streptomycin (GIBCO Life Technologies) and 10 μM retinoic acid (Sigma Aldrich). Primary cortical neurons were obtained from postnatal WT C57BL/6 J mouse (P0) using the Papain Dissociation System (Worthington Biochemical Corporation), plated in Neurobasal medium (GIBCO Life Technologies) supplemented with 5% (v/v) FBS (GIBCO Life Technologies), 2% B27 supplement (GIBCO Life Technologies), 0.5 mM L-glutamine (GIBCO Life Technologies), 100 U/mL penicillin, 100 μg/mL streptomycin (GIBCO Life Technologies) and cultured for 14 days under 5% CO_2_ at 37 °C. On the seventh day of culture, half of the supplemented Neurobasal medium was replaced with fresh medium.

### HiPSC culture and differentiation

GM23404*B iPSCs were thawed and subsequentially cultured according to the NIGMS Human Genetic Cell Repository, Coriell Institute for Medical Research (NJ, USA) guidelines for handling human iPSCs. Briefly, GM23404*B iPSCs were seeded on Murine Embryonic Fibroblasts (MEFs) and cultured with the human iPSC growth medium (DMEM/F12 Glutamax supplemented, 20% (v/v) Knock-Out Serum Replacement (KOSR), 1:100 Non-Essential Amino Acids (NEAA), 50 µM 2-mercaptoethanol and 25 U/mL penicillin-streptomycin (Thermo Fisher Scientific) supplemented with 10 ng/mL human FGF-2 (Miltenyi Biotec). Cells were passaged once or twice a week (at almost 90% confluency) using TrypLE^TM^ Select 1X (Thermo Fisher Scientific) and cell scrapers (EuroClone). During passages, 10 µM StemMACS Y27632 (Rock inhibitor, Miltenyi Biotec) was added and removed after 24 h. Human iPSC growth medium was refreshed daily and always supplemented with fresh FGF-2. After 3 passages in these culture conditions, cells were adapted to new culturing conditions as described in Campostrini et al. [78]. Briefly, hiPSCs were seeded on vitronectin recombinant human protein (Thermo Fisher Scientific) and cultured in E8 medium (Thermo Fisher Scientific). Cells were passaged twice a week (at 90% confluency) using PBS (Thermo Fisher Scientific) containing 0.5 mM EDTA (Thermo Fisher Scientific); 5 µM StemMACS Y27632 was added during passaging and removed after 24 h. E8 medium was refreshed daily. The differentiation in cardiomyocytes (CMs) was induced as described in [78, 79]. Briefly, 1 × 10^5^ GM23404*B iPSCs per well and 1.5 × 10^5^ LUMC0020iCTRL-06 iPSCs per well were seeded on 6-well plates coated with 0.17 mg/mL growth factor-reduced Matrigel^®^ (Corning), with E8 medium and 5 µM StemMACS Y27632, the day before starting the differentiation (day 1). On day 0, the cardiac mesoderm was induced by switching E8 medium to the LI-BPEL medium (LI-BPEL composition is reported in [49]), supplemented with a mixture of cytokines: 20 ng/mL BMP4 (R&D Systems), 20 ng/mL Activin A (Miltenyi Biotec), and 1.5 µM CHIR99021 (Axon Medchem). After 3 days, LI-BPEL medium supplemented with 5 µM XAV939 (Tocris) was added. Medium was then replaced every 3 days with LI-BPEL until the CMs monolayer started to beat spontaneously (between days 9–12) and, 1–2 days after spontaneous contraction appearance, medium was substituted with lactate purification medium (DMEM without glucose and pyruvate, Thermo Fisher Scientific; 4 mM sodium-L-lactate, Sigma-Aldrich) was added. After 4 days, the lactate selection was stopped, and medium switched to LI-BPEL. CMs at days 20–23 of differentiation were used for the experiments.

Neural Progenitor Cells (NPCs) were differentiated from the same hiPSCs cell lines using STEMdiff^TM^ Neural Induction Medium (STEMCELL Technologies) with the addition of 0.2% (v/v) of SMADi Neural Induction Supplement (STEMCELL Technologies). According to the technical manual provided by the manufacturer, hiPSC were cultured for 18–21 days (6 passages, as described above) on Matrigel (Corning) and TeSR™-E8™ medium (STEMCELL Technologies) before starting the differentiation. At day 0, hiPSCs were seeded on Matrigel^®^ coated 6-well plates at the density of 2 × 10^6^ cells and cultured in the differentiation medium, which was changed every day. The completed differentiation in NPCs had required 20 days during which cells were passaged 3 times at 100% confluence. During the passaging, cells were washed with DMEM/F-12 containing 15 mM HEPES (STEMCELL Technologies), detached using ACCUTASE^TM^ (STEMCELL Technologies), and seeded on Matrigel^®^ coated 6-well plate at the density of 1 × 10^6^ cells in differentiation medium supplemented with 10 µM StemMACS Y27632.

### SDS-PAGE and western blotting analysis

Cells from each line were harvested, sedimented, washed once with cold PBS and lysed for 30 min on ice in an appropriate volume of ice-cold RIPA lysis buffer (50 mM Tris-HCl, 150 mM NaCl, 1% (v/v) NP-40, pH 7.5) supplemented with cOmplete™ EDTA-free Protease Inhibitor Cocktail (Merck). Lysates were sedimented at full speed in a microcentrifuge for 30 min at 4 °C, the soluble fractions transferred to clean tubes and the protein concentration determined by the BCA assay (Pierce™ BCA Protein Assay Kit, Thermo Fisher Scientific). Equal protein amounts were solubilized in Laemmli gel sample buffer (62.5 mM Tris-HCl, 2% (w/v) SDS, 10% (v/v) glycerol, 5% (v/v) 2-mercaptoethanol, 0.1% (w/v) bromophenol blue, pH 6.8) and separated electrophoretically by SDS-PAGE on 4–20% polyacrylamide gel (GenScript® ExpressPlusTM PAGE) using Tris-MOPS-SDS as running buffer (GenScript® Running Buffer Powder). Proteins were then transferred on nitrocellulose membranes (0.45 μM, BioRad), through a semi-dry Trans-Blot® TurboTM Transfer System (BioRad). Membranes have been blocked with 10% milk in Tris-buffered Saline (TBS) for 1 h at room temperature and incubated at 4 °C overnight with primary antibodies, as specified in Table 1. After incubation with horseradish peroxidase-conjugated goat anti-mouse or anti-rabbit IgG at room temperature for 1 h (Table 1), proteins were visualized using Immobilon® Forte Western HRP Substrate (Millipore) by Imager CHEMI Premium Detector (VWR). Reaction product levels were quantified by scanning densitometry using ImageJ software and normalized to those of β-actin, TOM20 or citrate synthase.

**Table 1:** List of antibodies used in this work.

Western blotting	Antibody	Supplier	Condition of use	
	Anti – β-actin	Santa Cruz; SC-8432	1:1000 in TBST 1 × 5% milk – 3 h, RT	
	Anti – TOM20	Santa Cruz; SC-11415	1:1000 in TBST 1 × 5% milk – 3 h, RT	
	Anti – Citrate synthase	Invitrogen; MA5-17264	1:1000 in TBST 1X – O/N, 4 °C	
	Anti – FXN	Proteintech; 14147-1-AP	1:500 in TBST 1 × 5% milk – O/N, 4 °C	
	Anti – NDUFS1	Santa Cruz; SC-271510	1:500 in TBST 1X – O/N, 4 °C	
	Anti – NDUFS8	Santa Cruz; SC-515527	1:500 in TBST 1X – O/N, 4 °C	
	Anti – NDUFS7	Invitrogen; PA5-106367	1:500 in TBST 1X – O/N, 4 °C	
	Anti – NDUFV1	Invitrogen; PA5-21426	1:500 in TBST 1X – O/N, 4 °C	
	Anti – NDUFV2	Santa Cruz; SC-271620	1:500 in TBST 1X – O/N, 4 °C	
	Anti – SDHB	Santa Cruz; SC-59688	1:1000 in TBST 1X – O/N, 4 °C	
	Anti – UQCRFS1	Santa Cruz; SC-271609	1:500 in TBST 1X – O/N, 4 °C	
	Anti – ATP5A	Abcam; ab14748	1:4000 in TBST 1 × 5% milk—O/N, 4 °C	
	Anti – Vinculin	Sigma Aldrich; V9264	1:1000 in TBST 1X – O/N, 4 °C	
	Anti – Lamin B1	Santa Cruz; SC-6216	1:1000 in TBST 1 × 5% milk—O/N, 4 °C	
	Anti – Oct-3/4	Santa Cruz; SC-5279	1:500 in TBST 1X—O/N, 4 °C	
	Anti – Cardiac Troponin T	Abcam; ab45932	1:1500 in TBST 1X – 1 h, RT	
	Anti – Nestin	Santa Cruz; SC-23927	1:500 in TBST 1X – O/N, 4 °C	
	Anti – FLAG epitope	Invitrogen; 740001	1:750 in TBST 1X – O/N, 4 °C	
	Anti – mouse peroxidase	Sigma Aldrich; A4416	1:10 000 in TBST 1X – 1 h, RT	
	Anti – rabbit peroxidase	Sigma Aldrich; A0545	1:20 000 in TBST 1X – 1 h, RT	
	Anti – goat peroxidase	Sigma Aldrich; A5420	1:80 000 in TBST 1X – 1 h, RT	
IF / PLA	Antibody	Supplier	Condition of use for each cell line	
	Anti – Nestin	Santa Cruz; SC-23927	1:75 in 10% FBS in PBS—O/N, 4 °C	
	Anti – FXN	Proteintech; 14147-1-AP	LCLs	1:50 in 1% BSA in PBS—O/N, 4 °C
	Anti – MT-ND1 (CI)	Invitrogen; 43-8800	CMs / NPCs	1:50 in 10% FBS in PBS—O/N, 4 °C
	Anti – SDHB (CII)	Invitrogen; 459230	SH-SY5Y	1:50 in 5% FBS in PBS—O/N, 4 °C
	Anti – UQCRFS1 (CIII)	Santa Cruz; SC-271609	Mouse neurons	1:50 in 0.2% BSA, 10 mM glicine, 0.4% donkey normal serum, 0.02% Triton X-100 in PBS – O/N, 4 °C
	Anti – β-III-Tubulin	Synaptic Systems; 302306	SH-SY5Y	1:50 in 5% FBS in PBS—O/N, 4 °C
			Mouse neurons	1:200 in 0.2% BSA, 10 mM glicine, 0.4% donkey normal serum, 0.02% Triton X-100 in PBS—O/N, 4 °C
	Anti – chicken Alexa Fluor^TM^ 647	Invitrogen; A-21449	SH-SY5Y	1:200 in 5% FBS in PBS – 1 h, RT
			Mouse neurons	1:200 in 0.2% BSA, 10 mM glicine, 0.4% donkey normal serum, 0.02% Triton X-100 in PBS—1 h, RT
	Anti – mouse Alexa Fluor^TM^ 568	Invitrogen; A-11031	1:500 in 0.1% Tween-20 in PBS—1 h, RT	
	Anti – rabbit Alexa Fluor^TM^ 488	Invitrogen; A-11034	1:500 in 0.1% Tween-20 in PBS—1 h, RT	

### Co-Immunoprecipitation (Co-IP) assay

For Co-IP experiments, 30 × 10^6^ cells from the control LCL (i.e., GM07533) were collected, sedimented and washed once with cold PBS. The cell pellet was resuspended in an ice-cold Co-IP lysis buffer optimized to preserve weak protein-protein interactions (50 mM HEPES, 0.1% (v/v) Tween-20, 1% (v/v) Triton X-100, 10% (v/v) glycerol, pH 7.2) supplemented with cOmplete™ EDTA-free Protease Inhibitor Cocktail (Merck) and Halt™ Phosphatase Inhibitor Single-Use Cocktail (Thermo Fisher Scientific). Cells were kept on ice for 30 min, and the lysate was periodically and gently resuspended. The supernatant was then isolated by centrifugation at 12,000 × *g* for 15 min and transferred to a clean tube. Pre-clearing was performed mixing the soluble fraction with 50 µL of Protein-A Agarose beads (Roche), previously washed and equilibrated twice in ice-cold PBS and once in ice-cold Co-IP lysis buffer. This step was performed with gentle rotation, for 45 min at 4 °C, and the pre-cleared sample was recovered by centrifugation (300 × *g*, 1 min, 4 °C) and quantified by the BCA assay (Pierce™ BCA Protein Assay Kit, Thermo Fisher Scientific). For Co-IP assay, 5 mg of pre-cleared lysate was incubated with 2.5 μg of anti-FXN antibody (Proteintech; 14147-1-AP) in gentle and constant rotation at 4 °C, overnight. After that, 75 μL of pre-equilibrated Protein-A Agarose beads (Roche) were added and incubated with constant and gentle rotation for 1 h at 4 °C. After removal of the flow through, the resin was washed 3 times in ice-cold PBS and resuspended in 75 μL of Laemmli gel sample buffer, heated on a heat block at 95 °C for 15 min at constant shaking (750 rpm) and sedimented at full speed in a microcentrifuge for 1 min at room temperature, in order to recover the supernatant containing the IP sample. Samples of the Co-IP were loaded on the same 4–20% SDS-PAGE gel and analyzed by western blotting analysis, as described above.

### Immunofluorescence (IF)

For IF experiments on hiPSC-derived cardiomyocytes, 75 000 cells/well were seeded on 13 mm diameter cover glasses in 24-well plates, to reach approximately 70% final confluence. The day after seeding, cells were gently washed with PBS to remove the culture medium, fixed with 4% PFA in PBS for 15 min, permeabilized with 0.1% (v/v) Triton-X 100 (Sigma) in PBS for 10 min and saturated with 10% (v/v) FBS (GIBCO Life Technologies) at room temperature for 1 h in gentle shaking, in order to prevent non-specific binding of primary and secondary antibodies.

For IF experiments on hiPSC-derived neural progenitor cells, 200,000 cells/well were seeded on 13 mm diameter cover glasses in 24-well plates, to reach approximately 70% final confluence. One day after seeding, cells were gently washed with DMEM/F-12 containing 15 mM HEPES (STEMCELL Technologies), fixed with 4% PFA in PBS for 15 min, permeabilized with 0.2% (v/v) Triton-X 100 (Sigma) in PBS for 10 min and saturated with 10% (v/v) FBS (GIBCO Life Technologies) at room temperature for 2 h in gentle shaking.

For IF experiments on retinoic acid-differentiated neuroblastoma SH-SY5Y, 50,000 cells/well were seeded on poly-L-lysine (Sigma-Aldrich) pre-treated 13 mm diameter cover glasses in 24-well plates to reach 70% final confluence. To promote the differentiation of SH-SY5Y neuroblastoma cells into neuronal-like cells, 10 μM retinoic acid (Sigma-Aldrich) was newly added to the medium every 48 h. After 7 days of treatment, cells were gently washed with PBS, fixed with 4% PFA in PBS for 15 min at 4 °C, incubated with 50 mM NH_4_Cl in PBS for 10 min at room temperature and then permeabilized with ice-cold MeOH for 10 min at −20 °C. Saturation was performed with 5% (v/v) FBS (GIBCO Life Technologies) at room temperature for 3 h in gentle shaking.

IF experiments on primary mouse neurons, isolated as previously described and seeded on 13 mm diameter cover glasses in 24-well plates (150,000 cells/well), were conducted as it follows. Cells were washed with Hank’s Balanced Salt Solution (HBSS), fixed with 4% PFA (pH 7.4) in PBS for 30 min at room temperature, permeabilized with 0.3% (v/v) Triton-X 100 in PBS for 5 min at room temperature and saturated with blocking buffer (1% (w/v) BSA, 50 mM glycine, 2% (v/v) donkey normal serum, 0.1% (v/v) Triton-X 100 in PBS) at room temperature for 2.5 h in gentle shaking.

All samples were incubated overnight at 4 °C with primary antibodies and for 1 h at room temperature with fluorescent secondary antibodies, performing both the incubations in a humidified chamber (the list of antibodies and the experimental conditions of their use are reported in Table 1). Additionally, the differentiation of hiPSC cells was assessed through a staining with Phalloidin-iFluor 647 (ab176759, Abcam) for cardiomyocytes and with an anti-Nestin primary antibody for neural progenitor cells. In the case of SH-SY5Y differentiated into neuronal-like cells and primary mouse neurons, β-III-Tubulin was chosen as neuronal marker. Then, cover slides were washed with PBS for three times, dried and mounted with DAPI mounting media (Sigma). Images were acquired by means of Zeiss LSM 900 confocal microscope at 63X magnification and ZEN Software at the Imaging facility of the Department of Biology, University of Padova, and ultimately processed with ImageJ Fiji software.

### Proximity Ligation Assay (PLA)

A Duolink® In Situ Red Starter Kit Mouse/Rabbit (DUO92101, Sigma-Aldrich) was used to perform PLA. hiPSC-derived cardiomyocytes and neural progenitor cells were incubated with the couple of primary antibodies as described above for the IF experiments. Before the PLA experiments, lymphoblastoid cells (LCLs) were adhered onto microscope slides according to a protocol adapted from Tsang M. and collegues [80]. Briefly, suspension cells were collected by centrifugation at 200 × *g* for 10 min and the pellet was resuspended with PBS at a concentration of 2 × 10^6^ cells/mL. In a 24-well plate, 500 µL of cell suspension (i.e., 1 × 10^6^ cells) was gently transferred drop by drop on 13 mm diameter cover glass and the culture plate was incubated stationary for 30 min at room temperature to ensure the sedimentation and adhesion of cells. Non-adherent cells were then removed by aspirating off the supernatant from each well and adherent LCLs were fixed with 4% PFA in PBS for 10 min, washed once with 500 µL PBS for 5 min and permeabilized with 0.5% (v/v) Triton-X 100 in PBS for 10 min. After a wash with PBS for 5 min, cells were saturated with 1% (w/v) BSA in PBS at room temperature for 1 h, in gentle shaking, and incubated with the selected primary antibodies (Table 1, Supplementary Material). HiPSC-derived CMs/NPCs, SH-SY5Y differentiated into neuronal-like cells and primary mouse neurons were prepared and incubated with the couple of primary antibodies as described above for the IF experiments (Table 1). To perform PLA, cells were washed twice with Buffer A® (DUO82046, Sigma-Aldrich), for 5 min each at room temperature, and subsequently incubated for 1 h at 37 °C in a pre-heated humidified chamber with secondary antibodies conjugated with complementary oligonucleotides, i.e. anti-rabbit PLUS® (DUO82002, Sigma-Aldrich) and anti-mouse MINUS® (DUO82004, Sigma-Aldrich), properly diluted 1:5 in the Duolink® Antibody Diluent (DUO82008, Sigma-Aldrich). After 3 washes in Buffer A® (5 min each, at room temperature), cells were incubated with DNA Ligase (DUO82029, Sigma Aldrich) properly diluted 1:5 in Ligation Buffer (DUO82009, Sigma-Aldrich), for 30 min at 37 °C in a pre-heated humidified chamber. Subsequently, cells were washed 3 times in Buffer A® (5 min each, at room temperature) and incubated for 100 min at 37 °C with DNA Polymerase (DUO82030, Sigma-Aldrich) diluted 1:5 in Amplification Buffer (DUO82011, Sigma Aldrich) containing red fluorescent-labeled oligonucleotides, for 100 min at 37 °C in a pre-heated humidified chamber and in the dark. Cells were washed with 1X Buffer B® (DUO82048) for 10 min and eventually once with 0.01X Buffer B® for 1 min protecting them from light. The slides were then mounted using Duolink In Situ Mounting Medium containing DAPI (DUO82040, Sigma-Aldrich) and stored at −20 °C. Images were acquired by means of Zeiss LSM 900 confocal microscope at 63X magnification and ZEN Software at the Imaging facility of the Department of Biology, University of Padova. Images were ultimately processed with ImageJ Fiji software to quantify the number of PLA dots/cell.

### EPR spectroscopy

Mitochondrial enriched fractions were obtained starting from 700 × 10^6^ cells for each LCL. Cells were collected, pelleted, resuspended in ice-cold PBS and homogenized with a precooled 30 mL glass/Teflon tissue grinder through 20 slow up-down strokes at 1800 r.p.m. After the addition of 250 mM sucrose, the cell homogenate was centrifuged at 600 × *g*, 10 min, 2 °C and the supernatant was centrifuged at 14,000 × *g* for 10 min at 2 °C. The pellet, containing the mitochondrial fraction, was gently resuspended with 50 µL of a 50% glycerol solution in PBS. The suspension was kept on ice and 200 μL transferred to a 3 mm (i.d.) x 4 mm (o.d.) EPR quartz tube. The samples in the EPR tubes were immediately frozen in liquid nitrogen and stored in liquid nitrogen until spectroscopic analysis. Each step of subcellular fractionations was checked by western blotting analysis, using antibodies against lamin B1, vinculin and ATP5A (Table 1). EPR experiments were performed with an ELEXSYS E580 spectrometer equipped with a SHQ cavity (from Bruker) and with an Oxford ESR900 cryostat and an Oxford ITC4 temperature controller; liquid helium was used as a coolant. Spectral parameters were: temperature, 15 K or 100 K (parameters used exclusively for 100 K experiments are reported in parentheses); X-band frequency, 9.38 GHz; averaged scans, 25; power, 5 mW (0.02 mW); modulation amplitude, 0.7 mT; modulation frequency, 100 kHz; time constant, 41 ms; conversion time, 41 ms; scan width, 100 mT (12 mT); points, 1024. The magnetic field was calibrated using a DPPH sample (g = 2.0036). The baseline of each spectrum was corrected using the spectrum of an EPR sample with the mitochondria resuspension buffer (50% glycerol (v/v) in PBS) to subtract the cavity/cryostat contributions; a minor background by frozen oxygen was corrected, if present. The spectra were normalized for the total mitochondrial protein concentration as determined by BCA assay (control = 33 μg/μL; FRDA1 = 32 μg/μL; FRDA2 = 31 μg/μL). The quantifications were obtained as the peak-to-peak intensity of the signals centered at g = 1.92 or g = 2.006 for cluster N2 and the radicals, respectively, evaluating the error as three times the baseline noise.

### Lentiviral vectors production and transduction

A plasmid containing the coding sequence of human frataxin precursor (pET-9b/FXN^1-210^) was kindly provided by Dr. Javier Santos (Department of Physiology and Molecular and Cellular Biology, University of Buenos Aires, Argentina). A pUC57 vector containing the *T. thermophilus* Nqo15 coding sequence fused at the 5′ end to the mitochondrial import sequence of human COQ6 and to the 3′ end to a FLAG epitope coding sequence (Nqo15^COQ6^) was purchased from GenScript Biotech B.V. (Leiden, Netherlands). The FXN^1-210^ and Nqo15^COQ6^ were individually transferred into a lentiviral expression plasmid (pLenti6/V5-DEST™ Gateway™ Vector, Invitrogen). The identity of the inserts was confirmed by DNA Sanger sequencing (AbiPrims 3500, Applied Biosystems). To generate lentiviral particles, plasmids were co-transfected with packaging vectors (ViraPower Packaging Mix, Invitrogen) into HEK293-FT cells using Lipofectamine 2000 (Life Technologies) in OptiMEM (GIBCO Life Technologies). After 24 h, the medium was changed with DMEM high-glucose supplemented with 1 mM sodium pyruvate, 6 mM L-glutamine, 10% (v/v) FBS, 100 U/mL penicillin G, 100 µg/mL streptomycin and 1% (v/v) MEM Non-Essential Amino Acids and cells were maintained in culture for additional 48 h. Culture supernatants were harvested, filtered, frozen and kept at −80°C until viral transduction experiments. The transduction of FRDA fibroblasts was performed seeding 30 000 cells/well in a 96-well Seahorse plate adding 75% (v/v) of the viral particles’ suspension and 6 μg/mL of polybrene (Sigma-Aldrich). Cell plate was then incubated under 5% CO_2_ at 37 °C for 48 h and, after additional 24 h in fresh medium (DMEM 2 mM glucose supplemented with 1 mM sodium pyruvate, 2 mM L-glutamine, 10 µM uridine, 10% (v/v) FBS, 100 U/mL penicillin and 100 µg/mL streptomycin), the expression of FXN^1-210^ and Nqo15^COQ6^ proteins in FRDA cells was confirmed by western blotting analysis with an anti-FLAG antibody.

### Oxygen consumption studies

The Oxygen Consumption Rate (OCR) of transduced FRDA fibroblasts was measured by means of SeahorseXFe96 Analyzer (Agilent technologies). At fixed time points, oligomycin (1 μM), FCCP (750 nM), rotenone (1 μM) and antimycin A (1 μM) were added. To exclude wells with a massive detachment of cells from the analysis, every plate well was observed using an optic microscope and, to normalize respiration rates, the protein content was quantified by BCA assay after cell lysis. Bioenergetic parameters were assessed as it follows: basal respiration was calculated by subtracting the non-mitochondrial respiration rate (*i.e*. OCR value after the incubation with antimycin) from the OCR value before the addition of oligomycin; maximal respiration is the difference between the OCR value observed upon the addition of FCCP and the non-mitochondrial respiration rate; ATP-linked respiration was obtaining as the difference between OCR before and after the addition of oligomycin; spare respiratory capacity is the difference between the maximal and basal respiration.

### Biocomputational analysis

Analyses and molecular graphics of protein structures reported in this work were performed using UCSF Chimera software, developed by the Resource for Biocomputing, Visualization, and Informatics at the University of California, San Francisco with support from NIH P41-GM103311.

### Statistical analysis

All numerical data, analyzed by GraphPad Prism, are expressed as mean ± SEM, unless otherwise stated. Statistical analysis and significance were performed and assessed as specified in the figure legends, with *p* ≤ 0.05 accepted as statistically significant.

### Supplementary information

Supplementary material

Original wb

aj-checklist

Supplementary figure 1

Supplementary figure 2

Supplementary figure 3

Supplementary figure 4

Supplementary figure 5

## Data Availability

All relevant data are included within this manuscript and in Supplementary material.
