# Correction: Human frataxin, the Friedreich ataxia deficient protein, interacts with mitochondrial respiratory chain

**DOI:** 10.1038/s41419-024-06459-2

**Published:** 2024-01-29

**Authors:** Davide Doni, Federica Cavion, Marco Bortolus, Elisa Baschiera, Silvia Muccioli, Giulia Tombesi, Federica d’Ettorre, Daniele Ottaviani, Elena Marchesan, Luigi Leanza, Elisa Greggio, Elena Ziviani, Antonella Russo, Milena Bellin, Geppo Sartori, Donatella Carbonera, Leonardo Salviati, Paola Costantini

**Affiliations:** 1 Department of Biology, University of Padova, 35121 Padova, Italy; 2 Department of Chemical Sciences, University of Padova, 35131 Padova, Italy; 3 Clinical Genetics Unit, Department of Women’s and Children Health, University of Padova, 35128 Padova, Italy; 4 Istituto di Ricerca Pediatrica (IRP) Città della Speranza, 35127 Padova, Italy; 5 Centro Studi per la Neurodegenerazione (CESNE), University of Padova, Padova, Italy; 6 Department of Molecular Medicine, University of Padova, 35121 Padova, Italy; 7 Veneto Institute of Molecular Medicine, 35129 Padova, Italy; 8 Department of Anatomy and Embryology, Leiden University Medical Center, 2333 ZA Leiden, The Netherlands; 9 Department of Biomedical Sciences, University of Padova, 35121 Padova, Italy

**Keywords:** Mitochondria, Mechanisms of disease

Correction to: *Cell Death and Disease* 10.1038/s41419-023-06320-y, published online 08 December 2023

In this article the authors family and given names have been interchanged:

Doni Davide, Cavion Federica, Bortolus Marco, Baschiera Elisa, Muccioli Silvia, Tombesi Giulia, d’Ettorre Federica, Marchesan Elena, Leanza Luigi, Greggio Elisa, Ziviani Elena, Russo Antonella, Bellin Milena, Sartori Geppo, Carbonera Donatella, Salviati Leonardo and Costantini Paola.

They should read:

Davide Doni, Federica Cavion, Marco Bortolus, Elisa Baschiera, Silvia Muccioli, Giulia Tombesi, Federica d’Ettorre, Elena Marchesan, Luigi Leanza, Elisa Greggio, Elena Ziviani, Antonella Russo, Milena Bellin, Geppo Sartori, Donatella Carbonera, Leonardo Salviati and Paola Costantini.

The original article has been corrected.

