# Supplementary material for: Human frataxin, the Friedreich ataxia deficient protein, interacts with mitochondrial respiratory chain
Source: Cell Death Dis. 2023 Dec 8;14(12):805. doi: 10.1038/s41419-023-06320-y (PMC10703789; doi:10.1038/s41419-023-06320-y)
Supplement: Supplementary file 1 — Supplementary material [file 41419_2023_6320_MOESM1_ESM.docx]

**SUPPLEMENTARY MATERIAL**

**Human frataxin, the Friedreich ataxia deficient protein, interacts with mitochondrial respiratory complex I**

Doni D et al.

**Supplementary figure legends**

**Supplementary Figure 1. Differentiation of hiPSC from a healthy and a FRDA patient into CMs and NPCs.** Immunofluorescence staining and western blotting analysis of control and FRDA cell lines. IF (on the left) was performed using Phalloidin (in magenta) and Nestin (cyan) as markers for the cardiac and neural progenitor differentiation, respectively. Nuclei were counterstained with DAPI (blue). Scale bars: 30 μm. For western blotting analysis (on the right), OCT-3/4, Troponin T and Nestin were chosen as stemness, cardiac and neural progenitor markers, respectively. Equal amounts of protein (*i.e.*, 30 µg) were loaded in each lane and β-actin was used as loading control.

**Supplementary Figure 2. Co-localization of frataxin and mitochondrial respiratory chain complexes I, II and III in healthy and FRDA hiPSC-derived CMs and hiPSC-derived NPCs.** Double immunofluorescence staining of FXN (green) and complexes I, II, or III (red) in healthy (on the left) and FRDA (on the right) hiPSCs-CMs and hiPSCs-NPCs, resulting from the overlap of fluorescent signals (in yellow). Nuclei were counterstained with DAPI (blue). Scale bars: 30 μm.

**Supplementary Figure 3. Comparative analysis of frataxin levels in healthy and FRDA hiPSC-derived CMs, hiPSCs-derived NPCs, LCLs and fibroblasts.** Western blotting analysis of FXN protein in whole lysates from all cell lines used in this work. Equal amounts of protein (30 µg for CMs and NPCs; 45 µg for LCLs and fibroblasts) were loaded in each lane. β-actin was used as loading control. Protein levels were quantified after normalization with β-actin and expressed as a percentage of control level. Reported data result from the mean of at least four independent experiments ± SEM.

**Supplementary Figure 4. Negative controls of PLA experiments on healthy and FRDA hiPSC-derived CMs / NPCs.** Representative confocal images of *in situ* proximity ligation assay (PLA) performed on healthy and FRDA CMs and NPCs in which primary (left panel) or secondary (right panel) antibodies were omitted. The images refer to controls in which we omitted *i*) the couple of antibodies anti-frataxin/anti-complex I (we added the secondary antibodies), ii) the two secondary antibodies (we added the primary antibodies anti-frataxin/anti-complex I). Similar results were obtained using anti-frataxin in combination with anti-complex II and anti-complex III antibodies. Nuclei were counterstained with DAPI (blue). Scale bars: 30 μm.

**Supplementary Figure 5. NDUFS1 protein levels are reduced in FRDA fibroblasts, hiPSC-derived CMs and hiPSC-derived NPCs.** Western blotting analysis of respiratory complex I NDUFS1 subunit in whole cell lysates from fibroblasts, hiPSC-CMs and hiPSCs-NPCs used in this work. Equal amounts of protein (*i.e.*, 35 µg) were loaded in each lane. Citrate synthase was used as mitochondrial loading control. Protein levels were quantified after normalization with citrate synthase and expressed as a percentage of control level for each cell line. Reported data result from the mean of at least three independent experiments ± SEM. Statistical significance was determined using unpaired t-test (*p ≤ 0.05, ***p ≤ 0.001 compared to healthy control).
