# Supplementary figures and images for: Human frataxin, the Friedreich ataxia deficient protein, interacts with mitochondrial respiratory chain
Source: Cell Death Dis. 2023 Dec 8;14(12):805. doi: 10.1038/s41419-023-06320-y (PMC10703789; doi:10.1038/s41419-023-06320-y)

**Figure 3B**

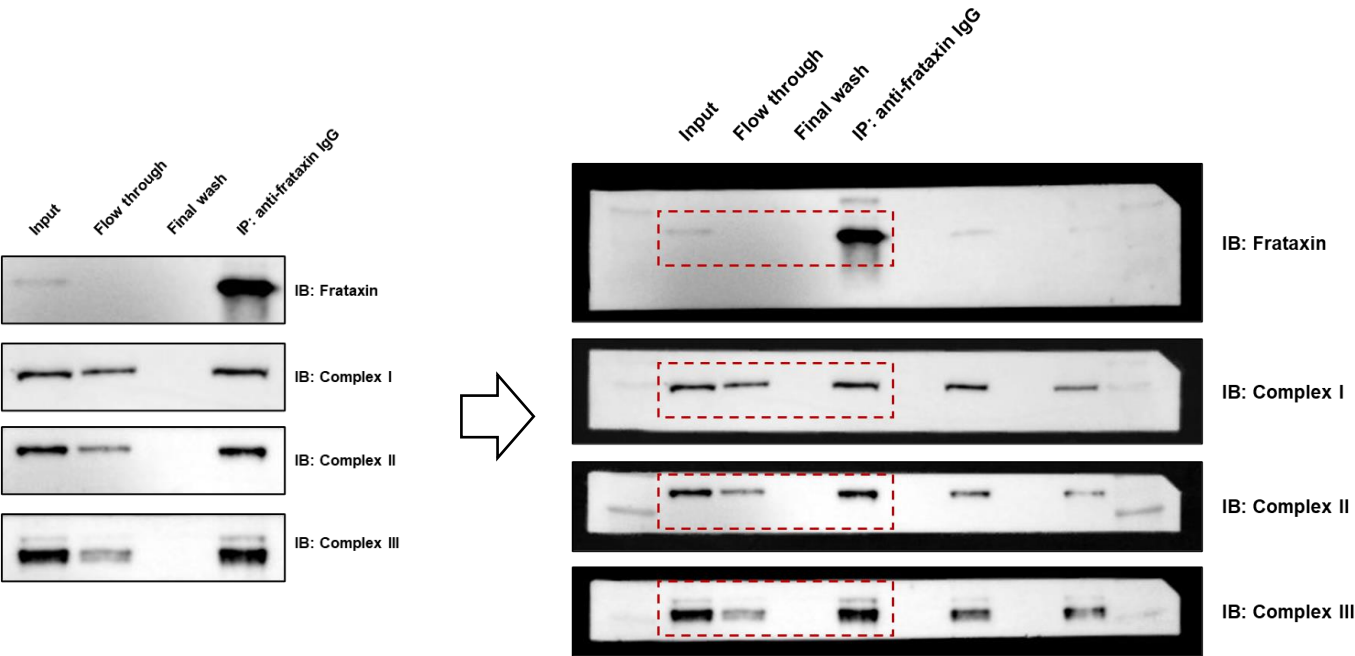

**Figure 4A**

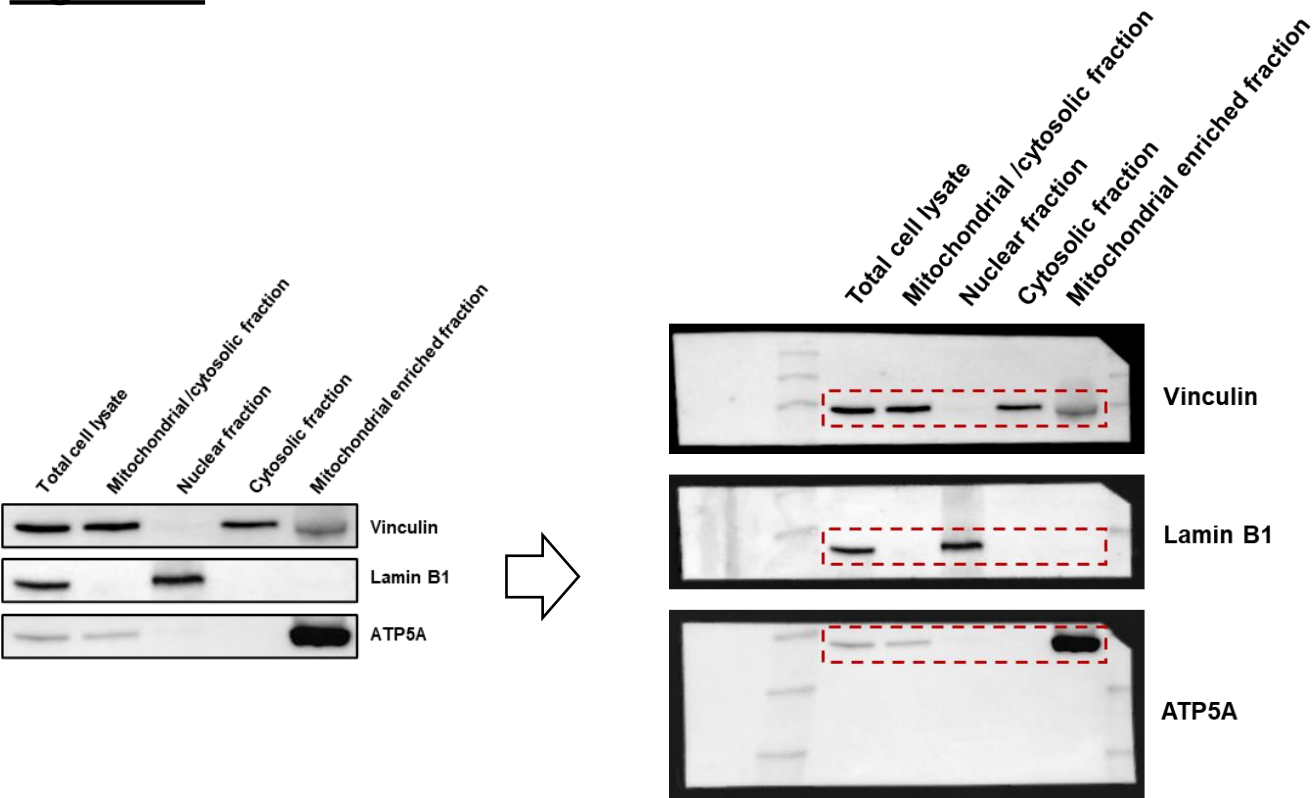

**Figure 5A**

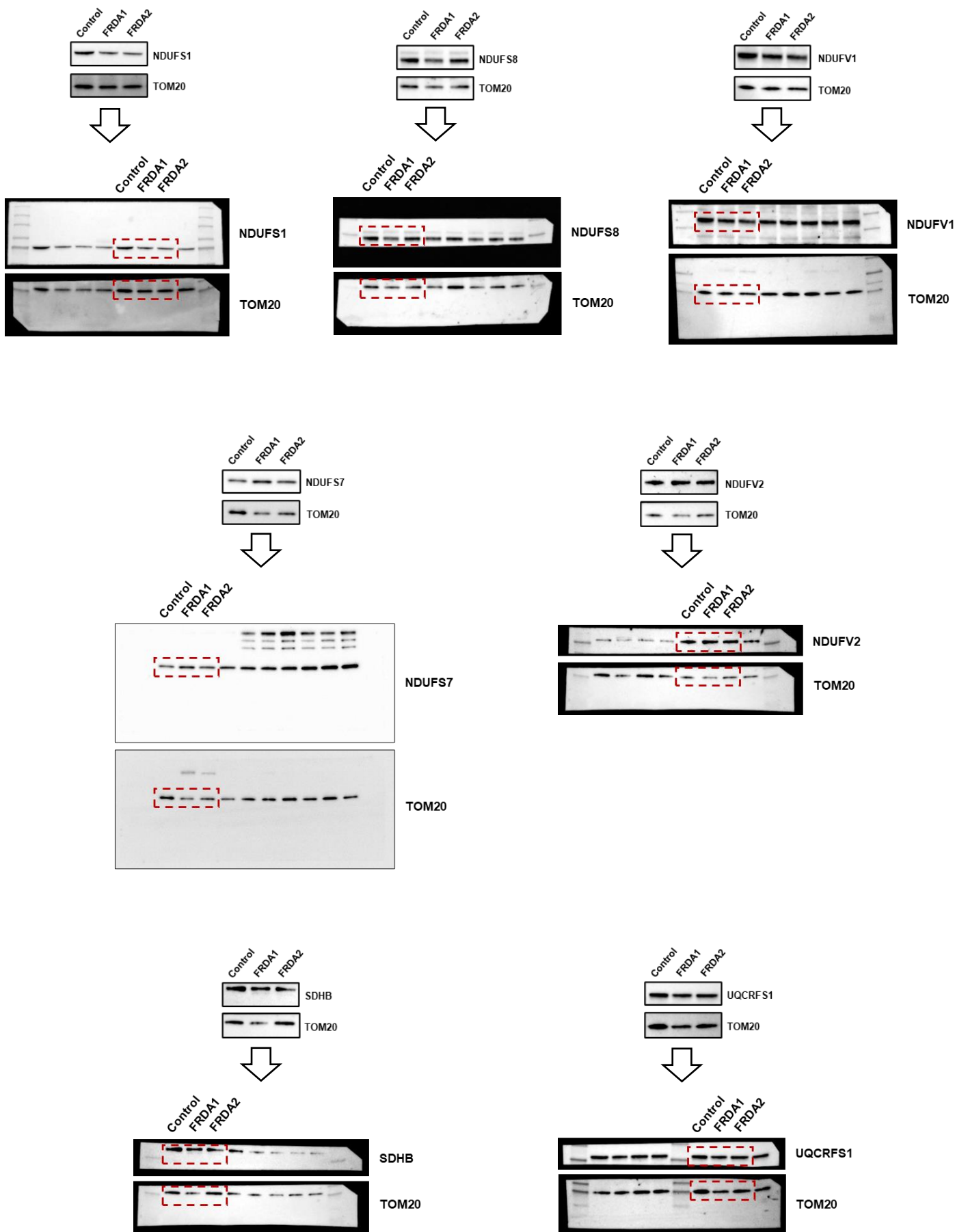

**Figure 6B**

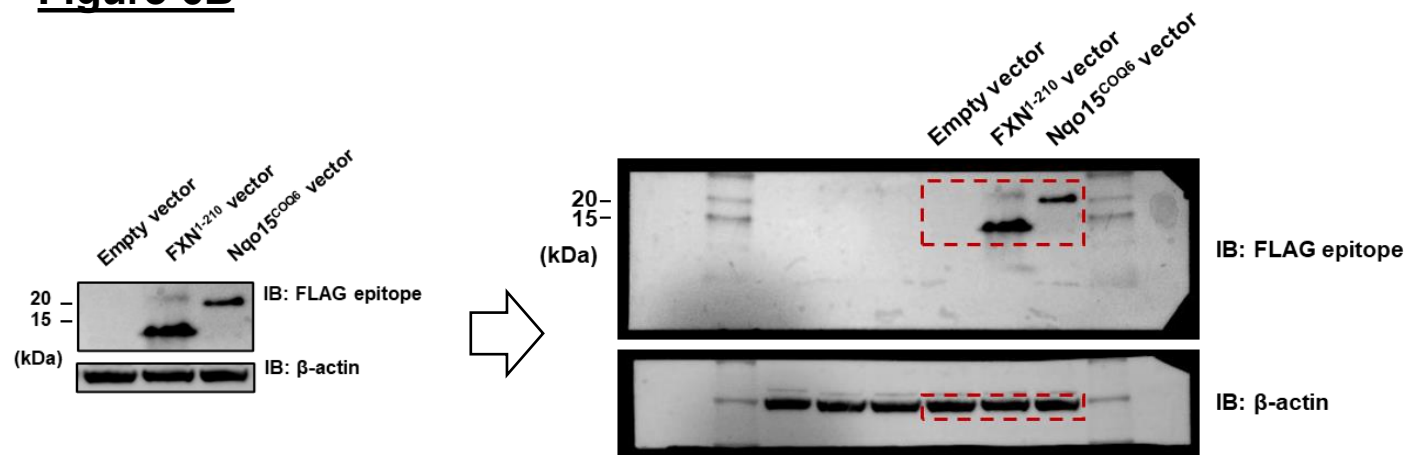

**Figure S1**

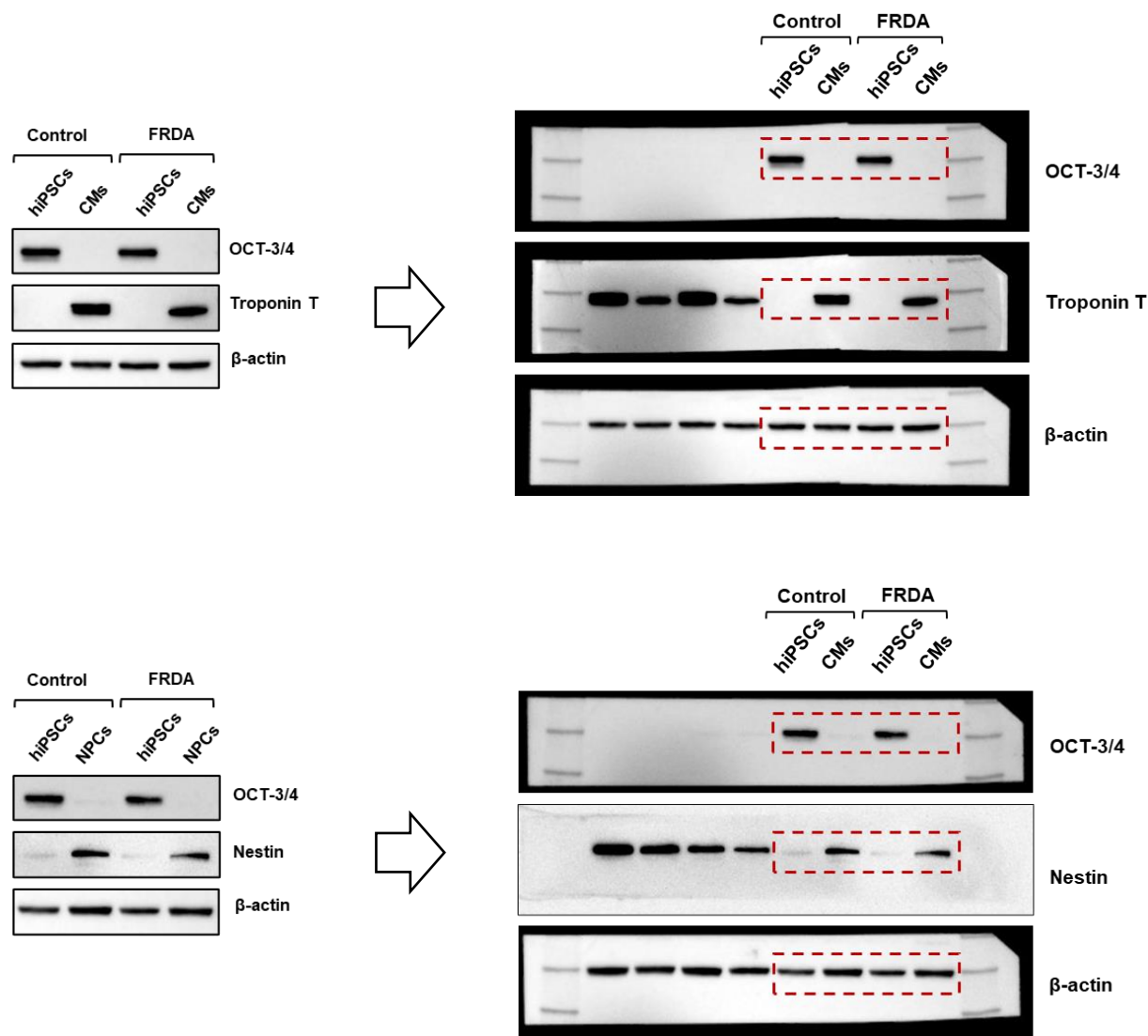

**Figure S3**

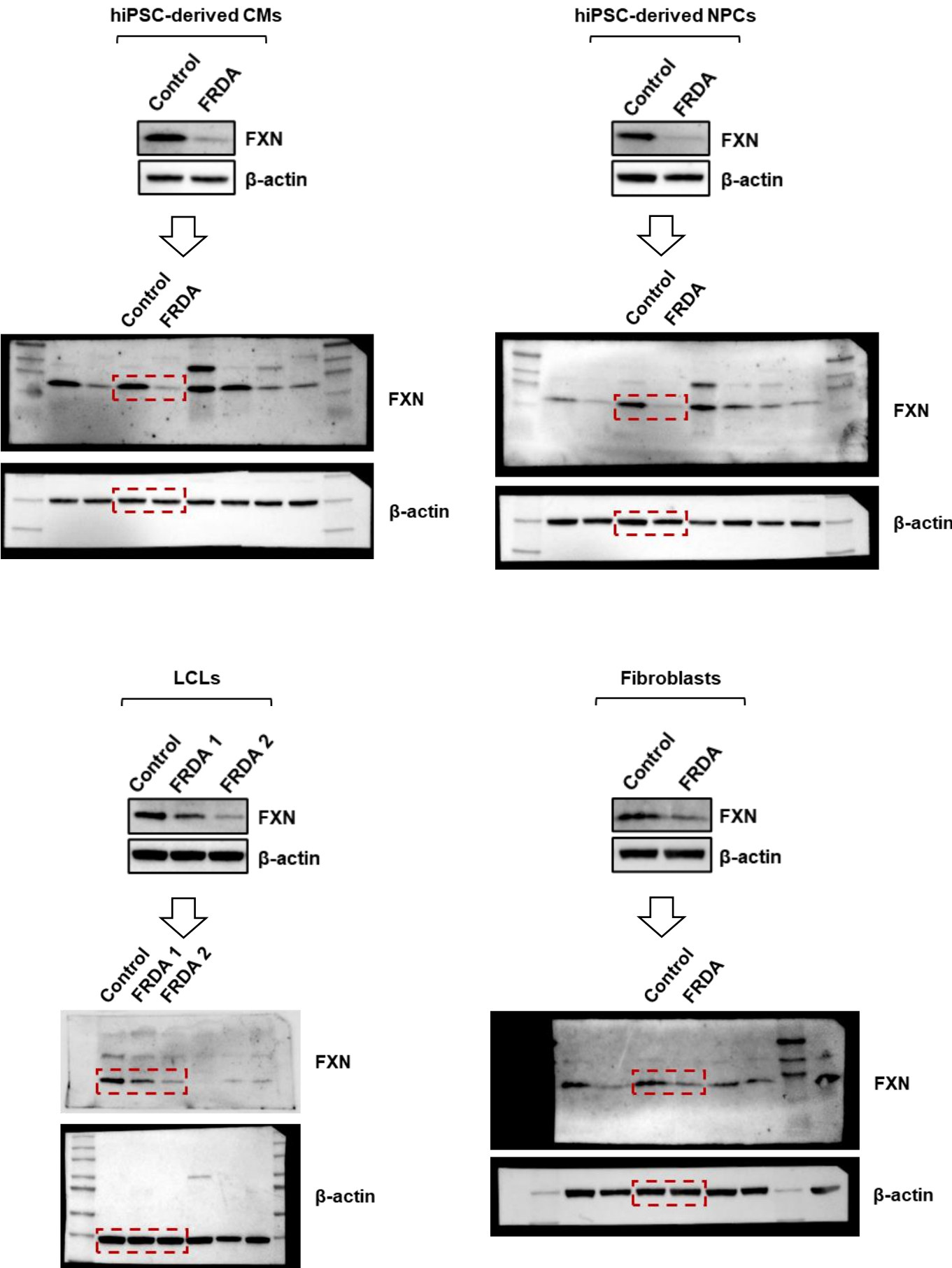

**Figure S5**

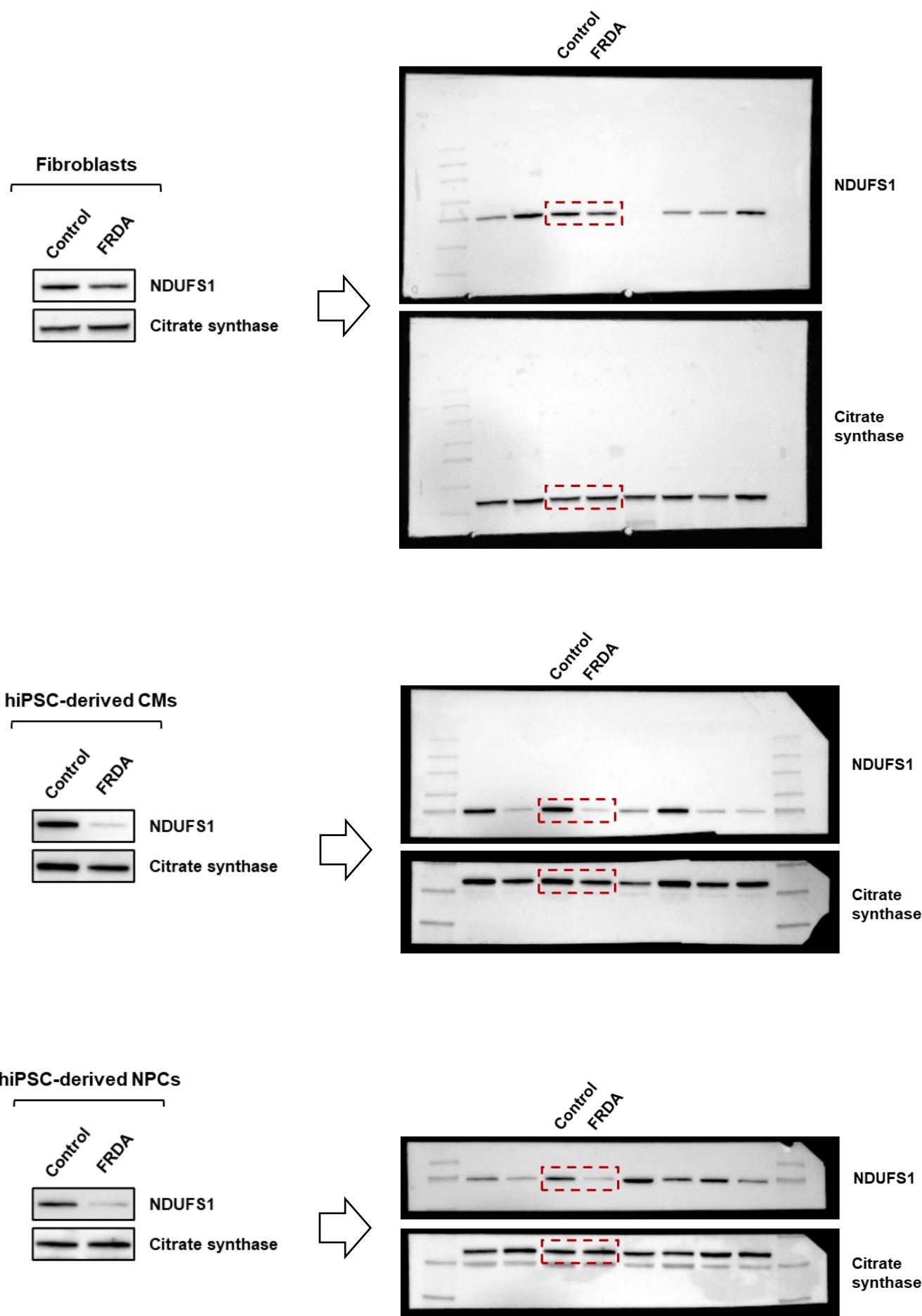

Supplement: Supplementary file 2 — Original wb [file 41419_2023_6320_MOESM2_ESM.pdf]

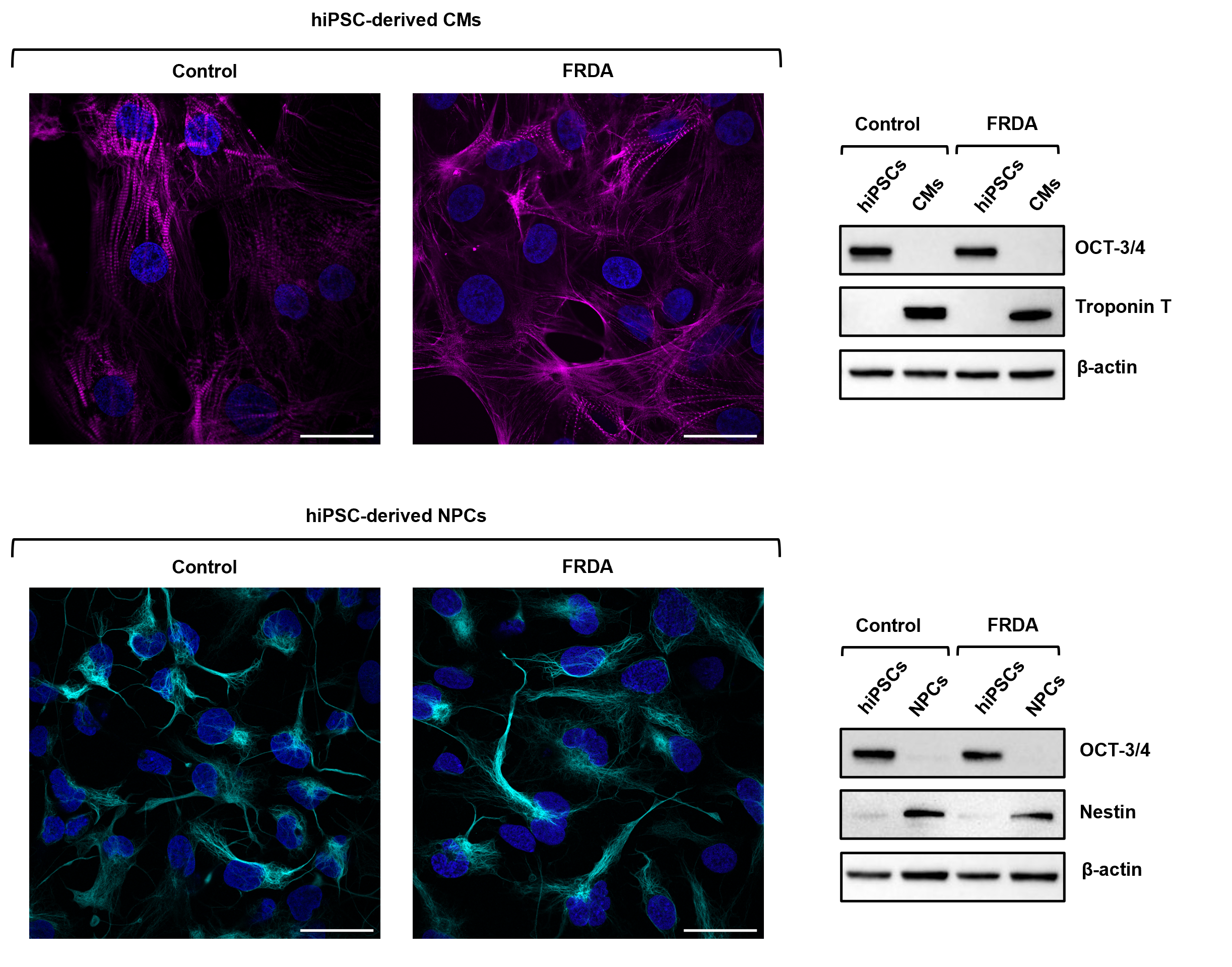

Supplement: Supplementary file 4 — Supplementary figure 1 [file 41419_2023_6320_MOESM4_ESM.png]

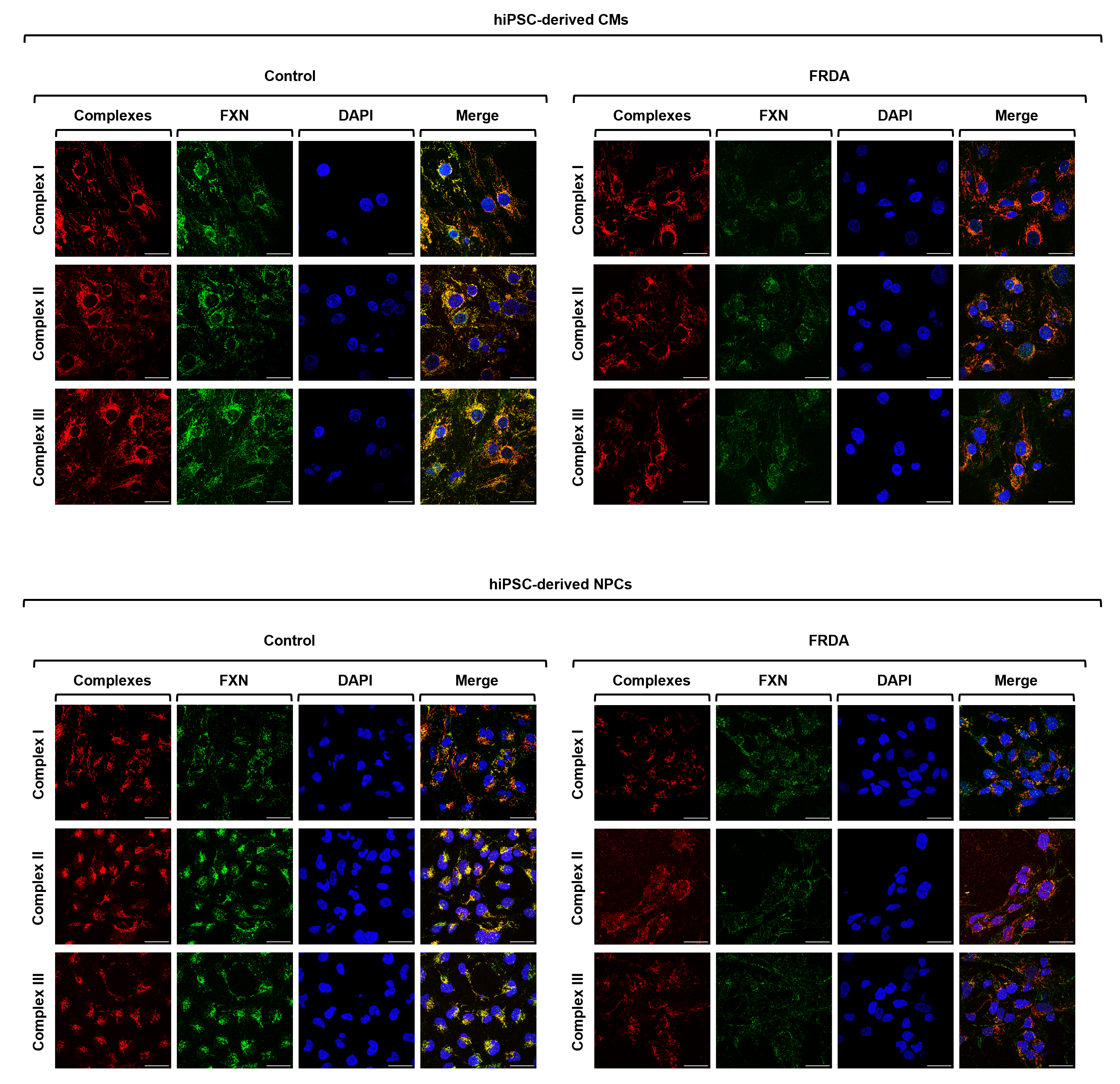

Supplement: Supplementary file 5 — Supplementary figure 2 [file 41419_2023_6320_MOESM5_ESM.png]

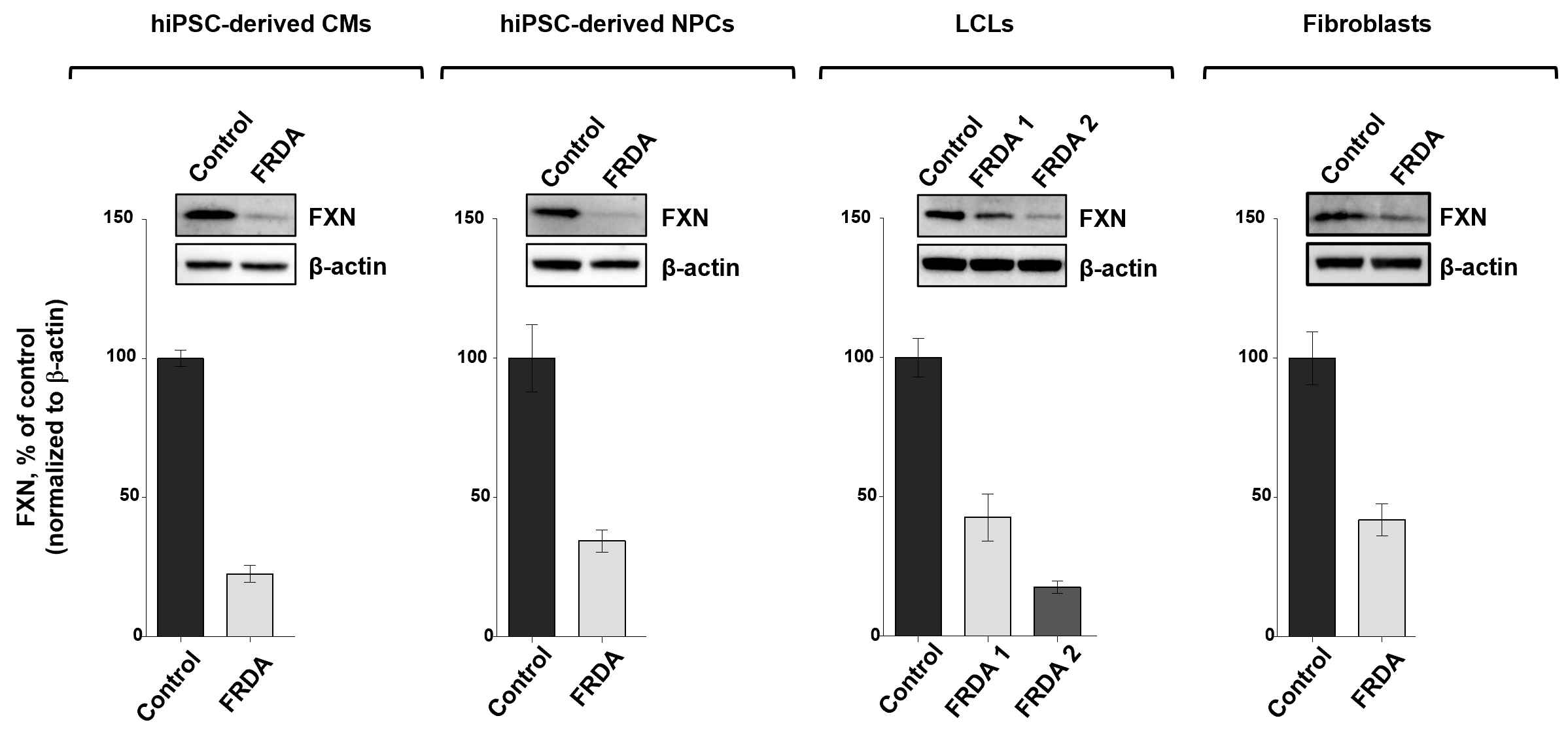

Supplement: Supplementary file 6 — Supplementary figure 3 [file 41419_2023_6320_MOESM6_ESM.png]

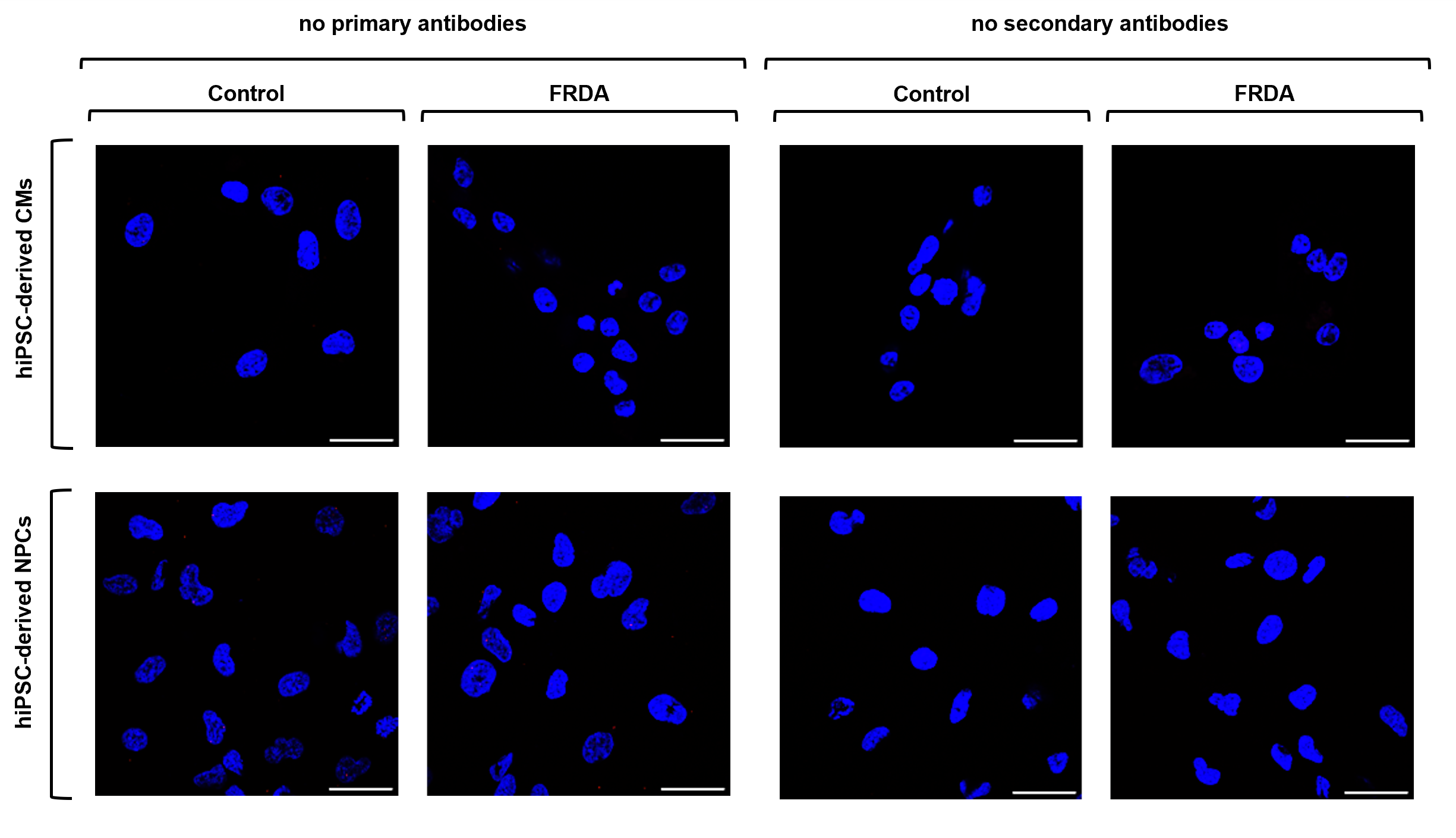

Supplement: Supplementary file 7 — Supplementary figure 4 [file 41419_2023_6320_MOESM7_ESM.png]

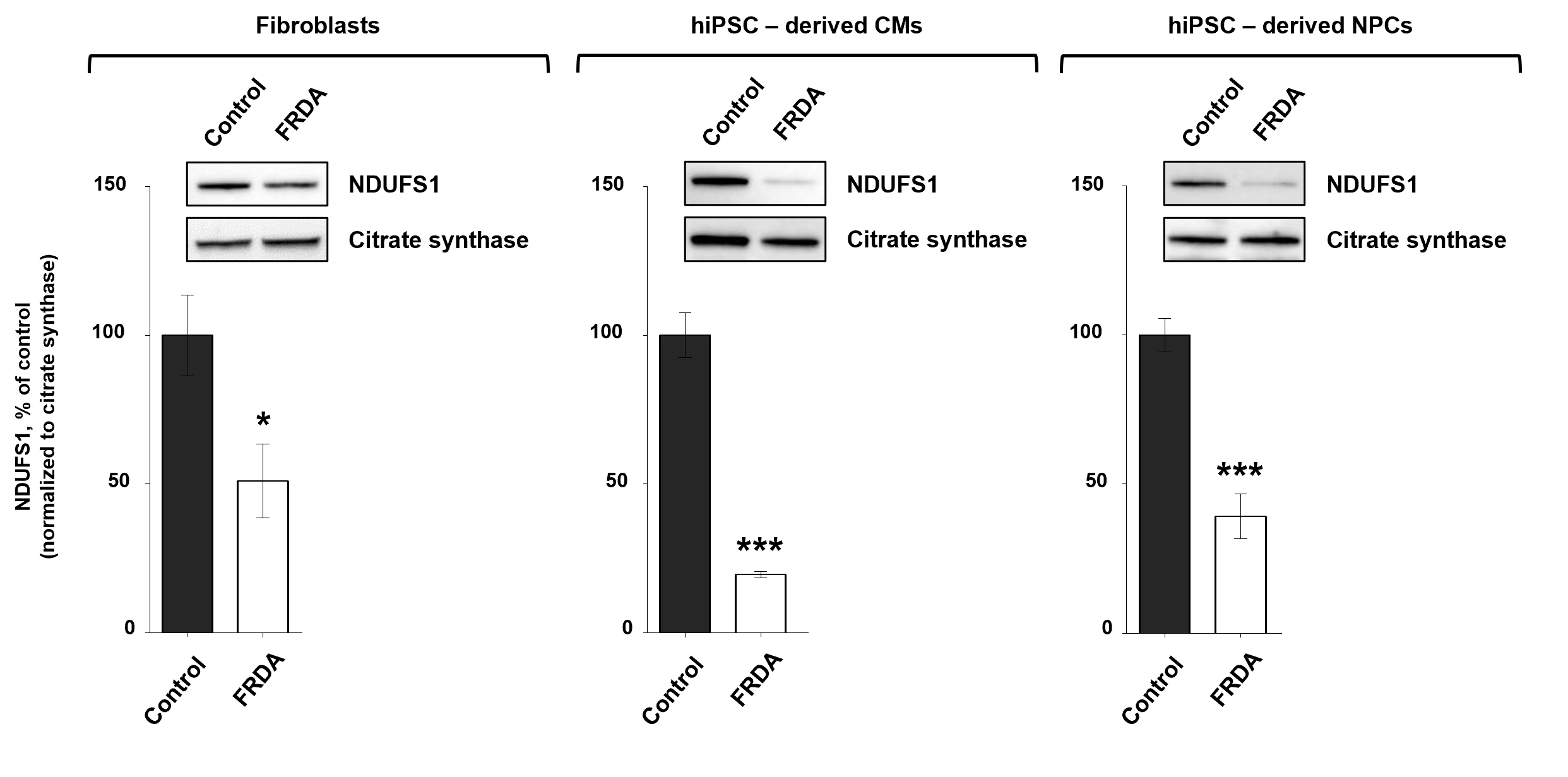

Supplement: Supplementary file 8 — Supplementary figure 5 [file 41419_2023_6320_MOESM8_ESM.png]
